# The development of imin-based tandem Michael–Mannich cyclocondensation through a single-electron transfer (SET)/energy transfer (EnT) pathway in the use of methylene blue (MB^+^) as a photo-redox catalyst†

**DOI:** 10.1039/d2ra01190e

**Published:** 2022-04-06

**Authors:** Farzaneh Mohamadpour

**Affiliations:** School of Engineering, Apadana Institute of Higher Education Shiraz Iran mohamadpour.f.7@gmail.com

## Abstract

A four-component green tandem approach for the metal-free synthesis of polyfunctionalized dihydro-2-oxypyrroles was devised using the Michael–Mannich cyclocondensation of amines, dialkyl acetylenedicarboxylaes, and formaldehyde. Photo-excited state functions generated from methylene blue (MB^+^) were employed as single-electron transfer (SET) and energy transfer (EnT) catalysts at ambient temperature in an ethanol solvent, employing visible light as a renewable energy source in the air atmosphere. This study aims to increase the usage of a non-metal cationic dye that is both inexpensive and widely available. Methylene blue is photochemically produced with the least amount of a catalyst due to its high yields, energy-effectiveness, high atom economy, time-saving features of the reaction, and operational simplicity. As a result, a variety of ecological and long-term chemical features are achieved. Surprisingly, such cyclization can be done on a gram scale, implying that the process has industrial potential.

## Abstract 2

A four-component green tandem approach for the metal-free synthesis of polyfunctionalized dihydro-2-oxypyrroles was devised using the Michael–Mannich cyclocondensation of amines, dialkyl acetylenedicarboxylaes, and formaldehyde.

## Graphical abstract

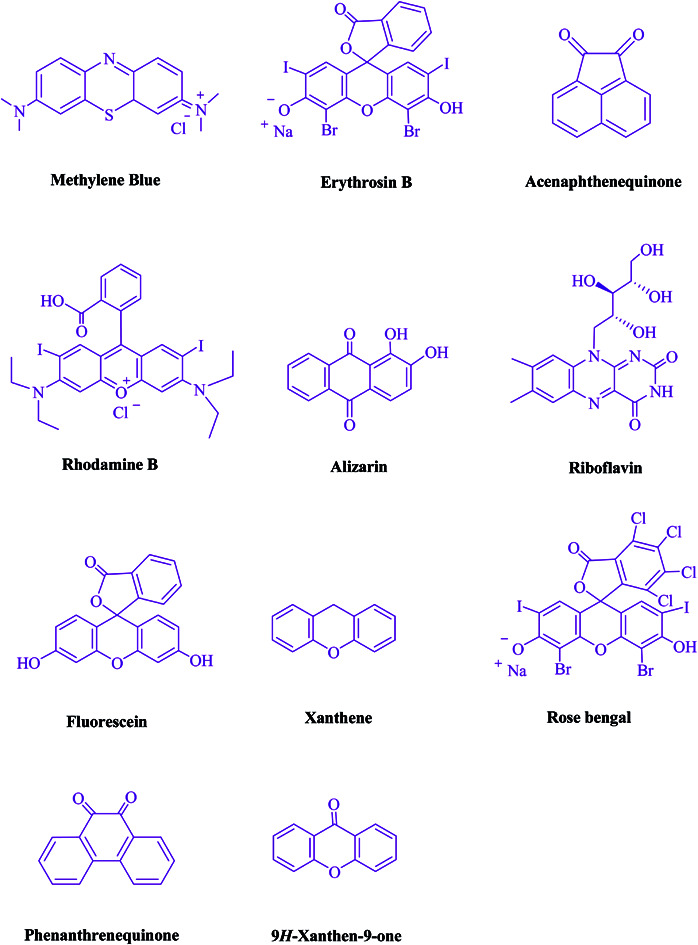

## Introduction

In recent years, the use of photo-redox catalysts in organic synthesis for the formation of C–C and C–heteroatom bonds *via* a single-electron transfer (SET)/photo-induced electron transfer (PET) pathway has exploded.^1^ They play an important role in a wide range of procedures, from small-scale to large-scale. As a result of technical improvements, various flow reactors^2^ utilizing visible light and dual photosensitized electrochemical processes^3^ have been created, resulting in more affordable, green, and efficient reactions. Methylene blue (MB^+^) was first synthesized in 1876, and its staining properties were later discovered. Methylene blue is a cationic dye that belongs to the thiazine dye family. Methylene blue has a range of medical operations. It has been demonstrated to be useful in treating methemoglobinemia and has impressive anti-malarial properties.^4–6^ One of the photophysiochemical properties of methylene blue is that MB^+^ has a singlet lifetime of *τ*_f_ ∼1.0 ns, as well as a 664 nm absorbance and molar absorbance (*ε* = 90 000).^7^ With a triplet lifespan of *τ*_f_ ∼32 μs,^8^ the triplet ^3^MB^+^* is a significantly more stable excited state.^1^

Furthermore, because visible light irradiation has enormous energy reserves, lower prices, and renewable energy sources, green chemists regard it as a reliable method for ecologically friendly organic chemical synthesis.^9,10^

As a result of their biological and pharmacological actions, biochemists and synthetic organic chemists have been attracted by the structures that make up pyrrole derivatives (Fig. S1† is provided in the ESI† file). Pyrrole derivatives have been reported in the literature as human cytomegalovirus protease (HCMV),^11^ human cytosolic carbonic anhydrase isozymes,^12^ PI-091,^13^ Oteromycin,^14^ cardiac cAMPphosphodiestrase,^15^ and most alkaloids have pyrrole rings.^16^

There have been some recent reports of polyfunctionalized dihydro-2-oxypyrroles being synthesized *via* multicomponent processes in the presence of various catalysts including as I_2_,^17^ glycine,^18^ AcOH,^19^ Cu(OAc)_2_.H_2_O,^20^ Fe_3_O_4_@nano-cellulose–OPO_3_H,^21^ BiFeO_3_ nanoparticles,^22^ nano-Fe_3_O_4_@SiO_2_/SnCl_4_,^23^ glutamic acid,^24^ graphene oxide,^25^ CoFe_2_O_4_@SiO_2_@IRMOF-3,^26^ 2,6-pyridinedicarboxylic acid,^27^ saccharin,^28^ tartaric acid,^29^ lemon juice,^30^ nano-H_3_PW_12_O_40_/Fe_3_O_4_@SiO_2_-Pr-Pi,^31^ UiO-66-SO_3_H,^32^ caffeine,^33^ nano-TiCl_4_/SiO_2_,^34^ Fe/MWCNTs,^35^ trityl chloride^36^ and EDDF.^37^ These methods have resulted in metal catalyst limitations, expensive reagents, harsh reaction conditions, monotonous unacceptable yields, environmental risks, workup processes, and long reaction times. Furthermore, separating a homogenous catalyst from the reaction mixture is difficult. Given the previous and our attempts to produce polyfunctionalized dihydro-2-oxypyrroles, the purpose of this study was to evaluate photocatalysts^38^ in green settings in order to produce these biologically active molecules. This research also makes it easy to use a low-cost, widely available metal-free cationic dye photo-redox catalyst. Methylene blue (MB^+^) is the product of the photochemical reaction described above. When the reaction is highly effective, simple, and mild, this is an effective one-pot technique.

## Materials and methods

### Characterization

The melting points of all compounds were determined using Electrothermal 9100 equipment. Furthermore, CDCl_3_ was used to record nuclear magnetic resonance, ^1^HNMR and ^13^CNMR spectra using a Bruker DRX-400, Bruker DRX-300, and Bruker DRX-100 Avance tool. The mass spectra were obtained employing a spectrometer from Agilent Innovation (HP) working at a 70 eV ionization potential. We bought the entire reagents from the chemical companies called Fluka (Buchs, Switzer-land), Acros (Geel, Belgium), and Merck (Darmstadt, Germany) and used them without additional purification.

### The entire procedure for preparing 5a-s

**Scheme 1:**
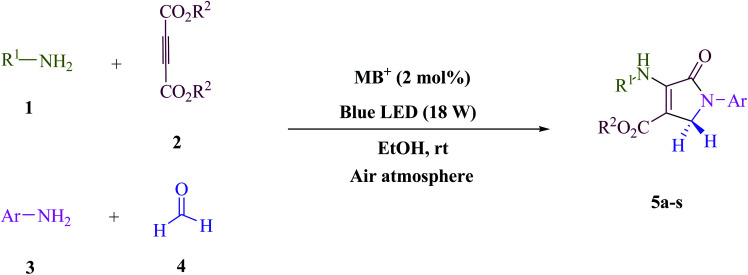
Polyfunctionalized dihydro-2-oxypyrroles synthesized.

In EtOH (3 mL), amine 1 (1.0 mmol) and dialkyl acetylenedicarboxylate 2 (1.0 mmol) were agitated for 15 min under blue light (LED) irradiation (18 W). After that, add amine 3 (1.0 mmol), formaldehyde 4 (1.5 mmol), and methylene blue (2 mol%) to the mixture (Scheme 1). At room temperature, the mixture was agitated. After finishing the reaction, the mixture was filtered (using thin-layer chromatography TLC), and the solid was washed with EtOH without column chromatographic separation to achieve pure chemicals. Even if we could make the aforementioned compounds utilizing gram scale methods, we wanted to explore if we could scale up to the degree that pharmaceutical process R&D required. In one experiment, 50 mmol aniline, 37.5 mmol formaldehyde, and 25 mmol diethyl acetylenedicarboxylate (DEAD) were mixed. The large-scale reaction went off without a hitch and took only 25 min to complete, with the product collected using typical filtration methods. This material's ^1^HNMR spectrum indicates that it is spectroscopically pure.

The products were classified based on their spectroscopic information (^1^HNMR, ^13^CNMR, and mass) and the detailed information can be found in the ESI.†

## Results and discussion

**Fig. 1:**
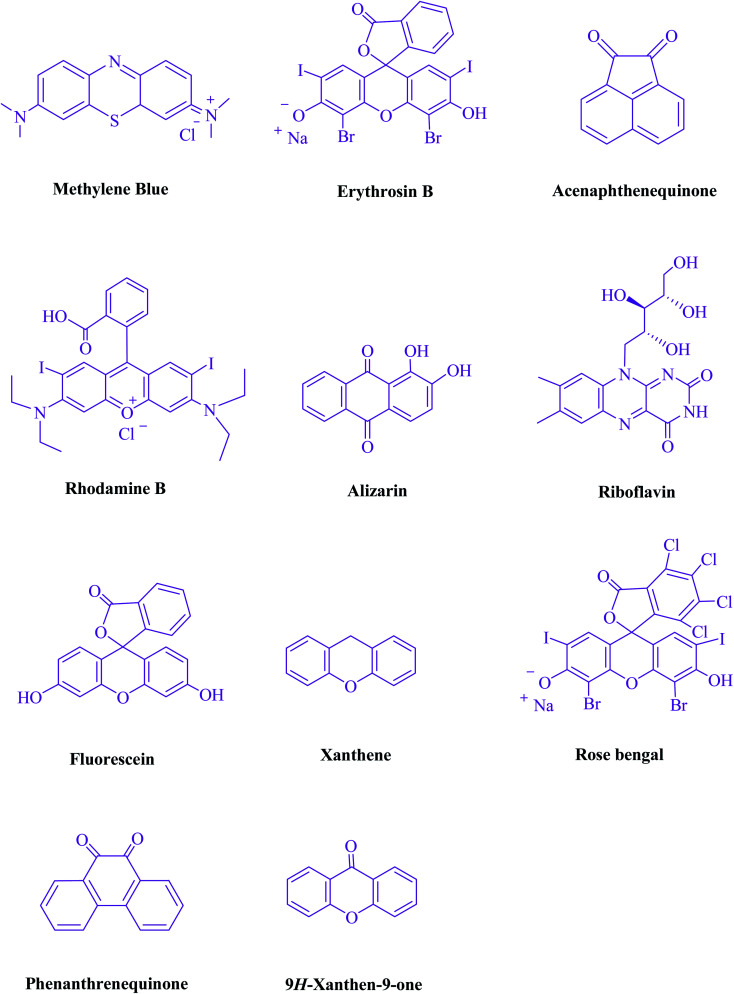
Photocatalysts were put to the test in this study.

To begin, the reaction of formaldehyde (1.5 mmol), aniline (2 mmol), and dimethyl acetylenedicarboxylate (DMAD) (1 mmol) in EtOH (3 mL) at room temperature under LED irradiation were examined. With no photocatalysts, there was a trace of 5a at rt in 3 mL EtOH for 40 min (Table 1, entry 1). To promote the reaction, methylene blue, erythrosin B, acenaphthenequinone, rhodamine B, alizarin, riboflavin, fluorescein, xanthene, rose Bengal, phenanthrenequinone, 9*H*-xanthen-9-one (Fig. 1) were all examined in identical conditions. The advancement of this reaction was seen in 42–95% yields while attaining the acceptable matched product 5a. Methylene blue fared better in such a response, according to the findings. Using 2 mol% MB^+^, the yield was increased to 95% (Table 1, entry 5). DMF, DMSO, THF, and toluene all exhibited lower product yields. The reaction rate and yield were improved in CH_3_CN, MeOH, EtOAc, H_2_O, and solvent-free conditions. The reaction was carried out in EtOH with outstanding yield and rate and a yield of 95% was achieved under identical conditions. To screen the yield, different light sources were used, demonstrating the effect of blue light. According to the test control, there was a miniscule of 5a without employing the light source. Visible light and MB^+^ are essential for the successful synthesis of product 5a, according to the findings. In addition, the enhanced settings were determined by irradiating blue LEDs of varying intensities (10, 12, 18, and 20 W). According to the researchers, the best results were obtained when blue LEDs (18 W) were used (More data is provided in Table S1† and Table S2† in the ESI† file). Under the right conditions, a wide range of substrates was investigated (Table 2 and Scheme 1). It's worth noting that the aniline substituent had no effect on the outcome of the reaction (Table 2). Halide substitutions were allowed under the reaction conditions. Reactions involving both electron-donating and electron-withdrawing functional groups went well in the current reaction state. All aliphatic and benzylic amines have a very high yield. Dimethyl acetylenedicarboxylate (DMAD) and diethyl acetylenedicarboxylate (DEAD) had comparable reaction patterns (Table 2). Table 2 also includes information on turnover number (TON) and frequency of turnover (TOF). The higher the TON and TOF numerical values are, the less catalyst is utilized and the higher the yield, and the catalyst becomes more effective as the value rises.

**Table 1:** Optimization table of photocatalyst for the synthesis of 5a^a^

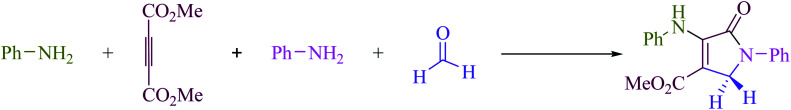				
Entry	Photocatalyst	Solvent (3 mL)	Time (min)	Isolated Yields (%)
1	—	EtOH	40	Trace
2	Methylene blue (0.5 mol%)	EtOH	25	42
3	Methylene blue (1 mol%)	EtOH	25	58
4	Methylene blue (1.5 mol%)	EtOH	25	84
**5**	**Methylene blue (2 mol%)**	**EtOH**	**25**	**95**

a Reaction conditions: EtOH (3 mL), blue LED (18 W), and different molar photocatalyst at room temperature, formaldehyde (1.5 mmol), aniline (2 mmol), and dimethyl acetylenedicarboxylate (DMAD) (1 mmol).

**Table 2:** For the production of polyfunctionalized dihydro-2-oxypyrroles, photoexcited MB^+^ was utilized as a photo-redox catalyst

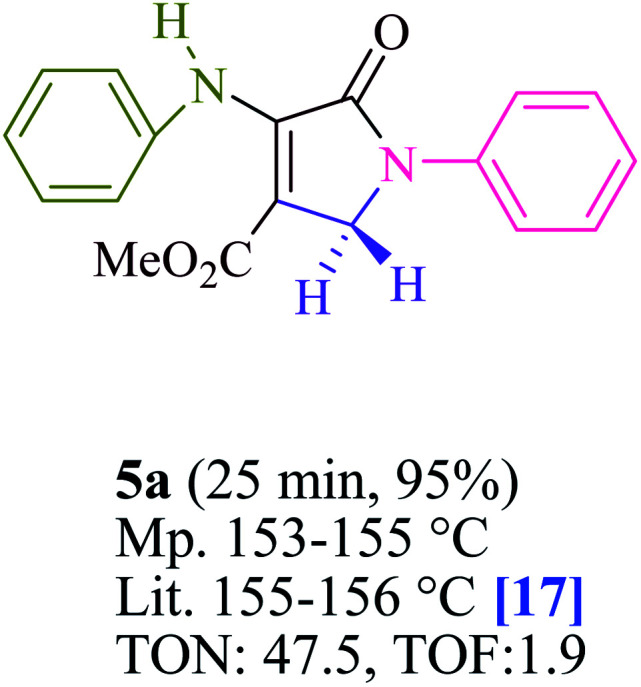	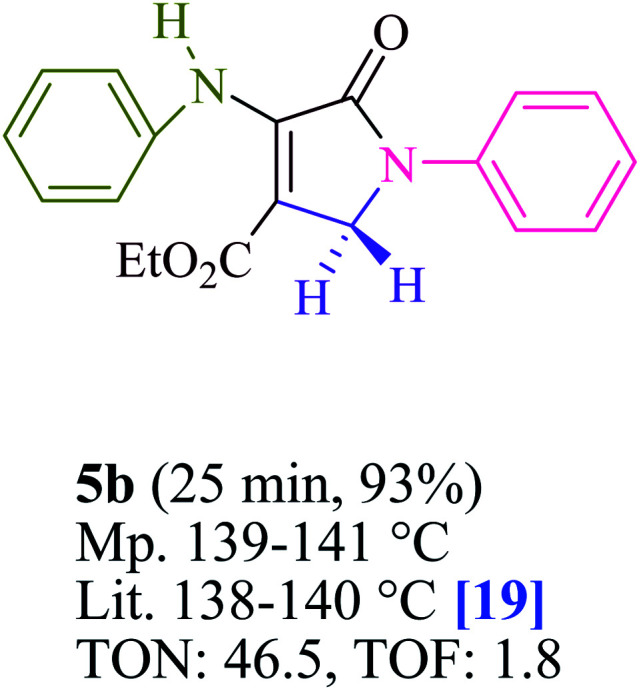
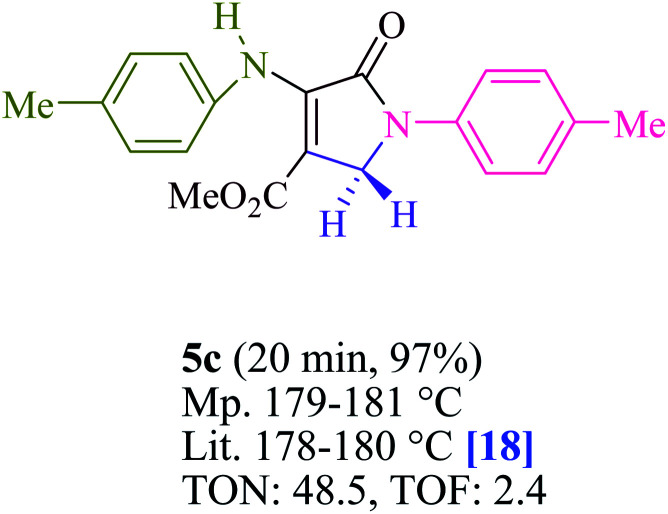	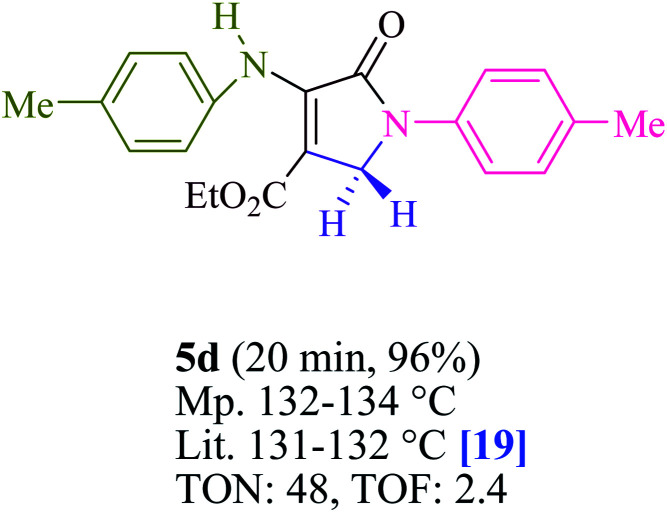
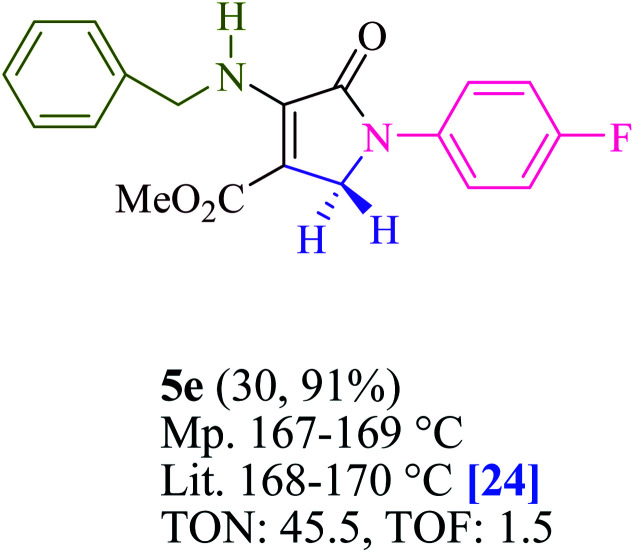	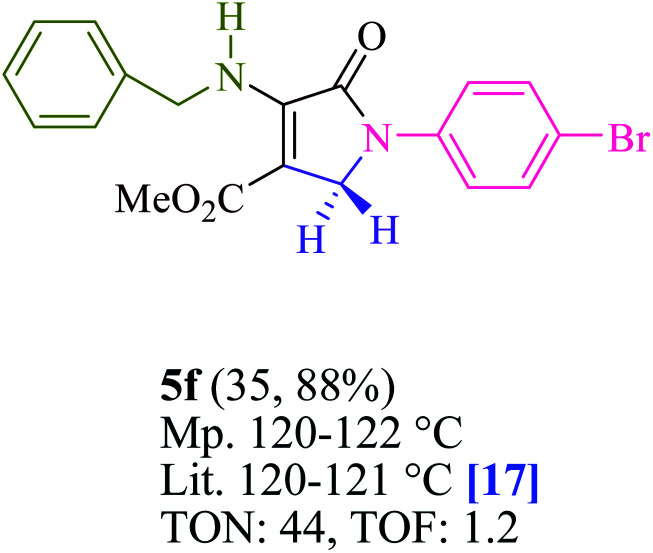
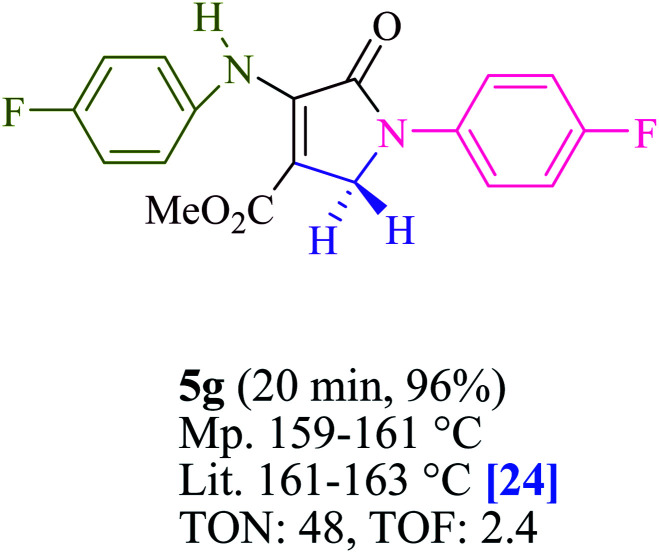	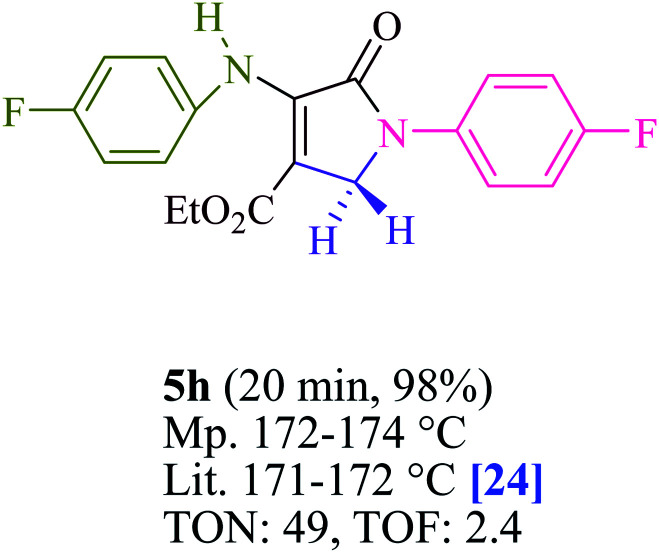
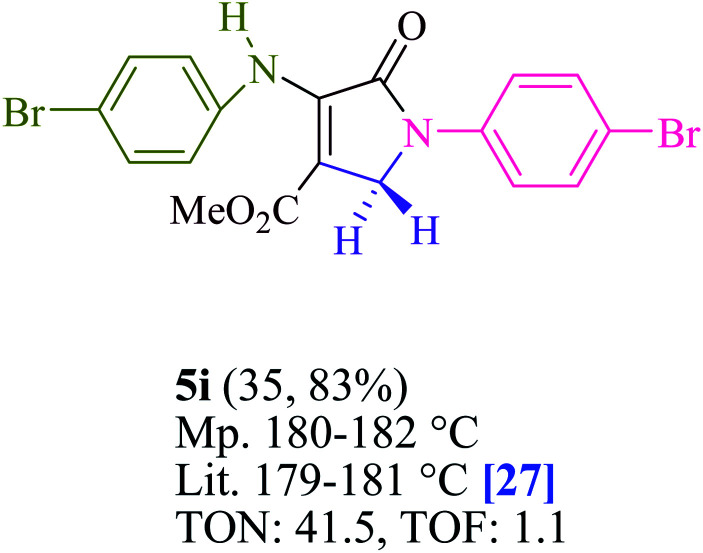	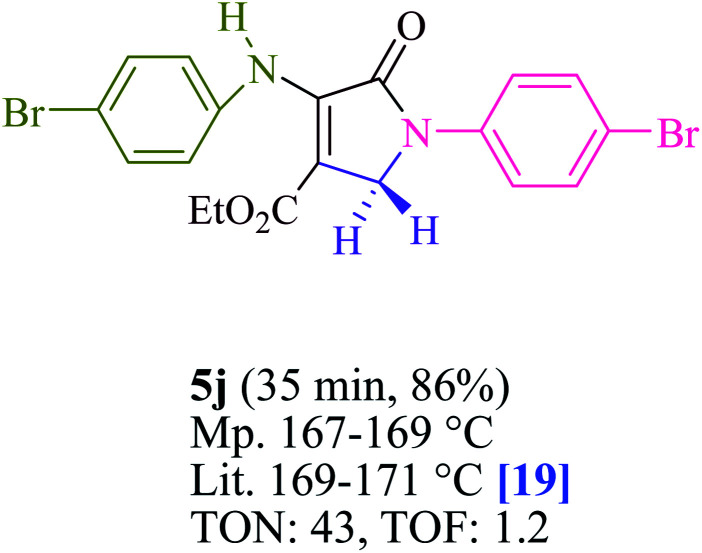
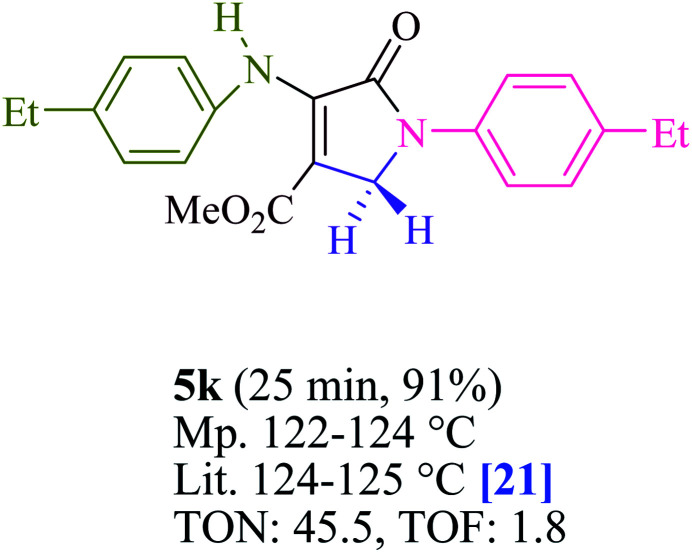	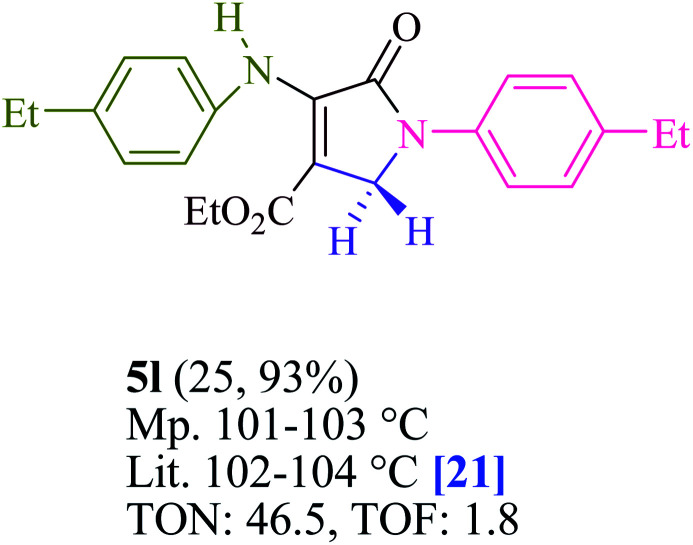
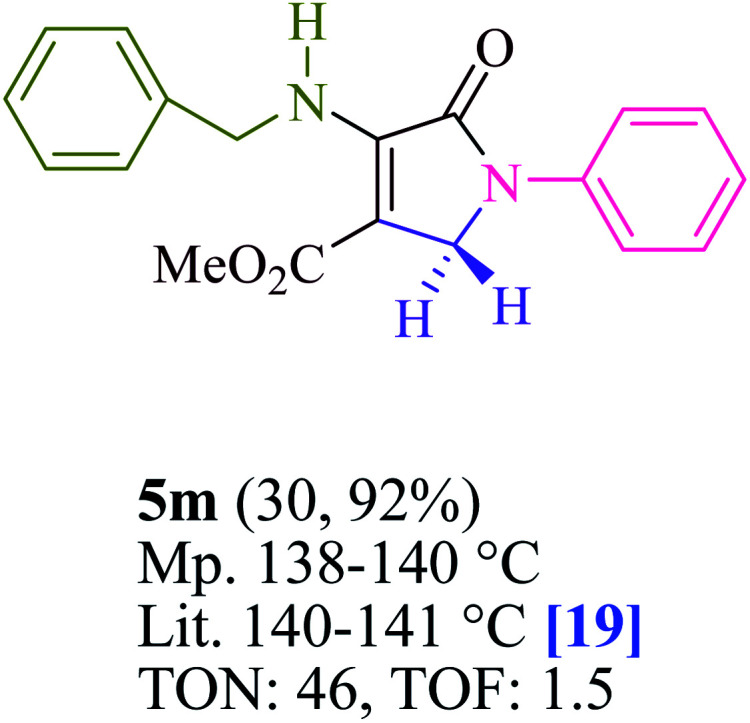	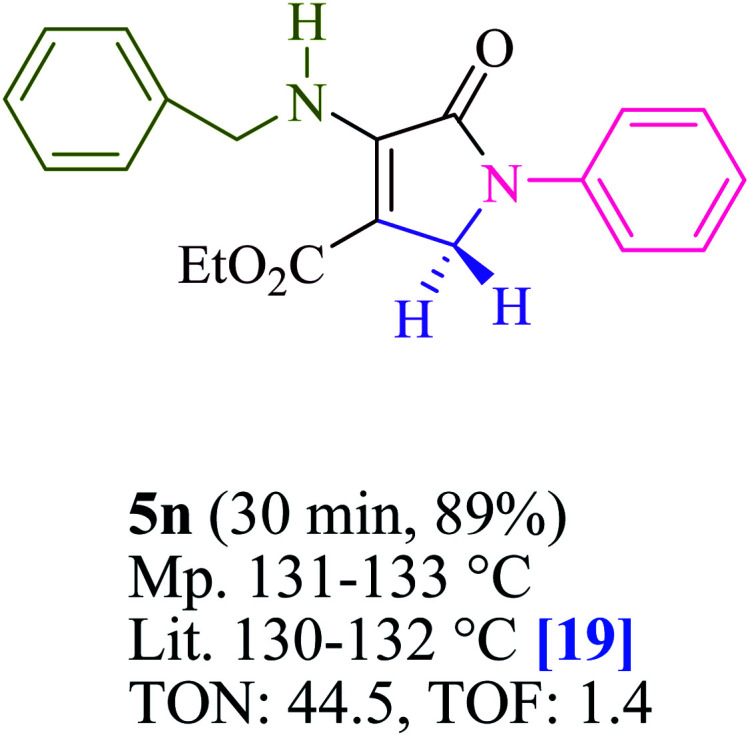
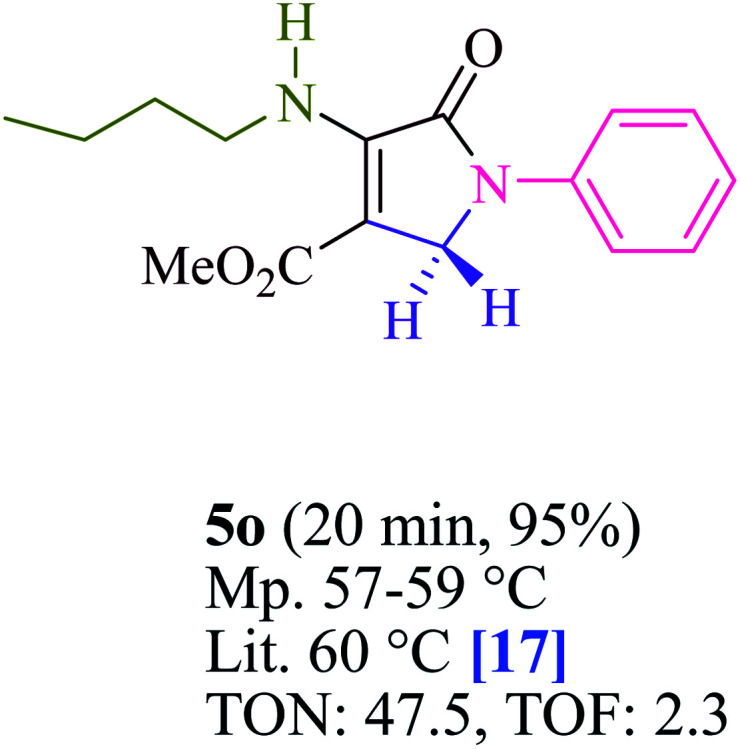	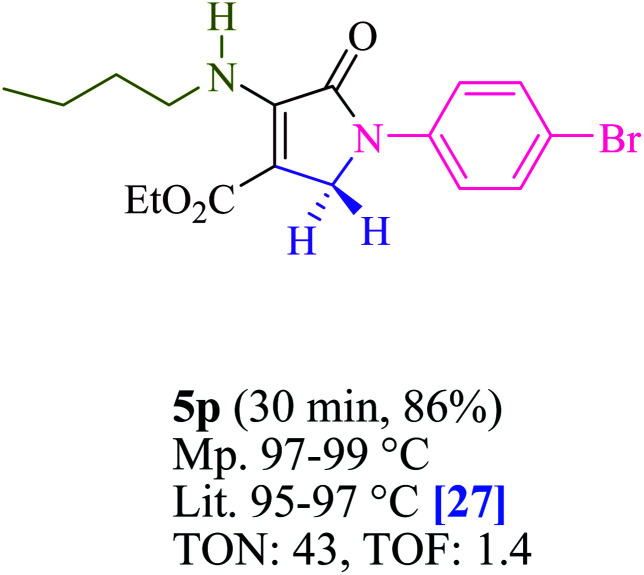
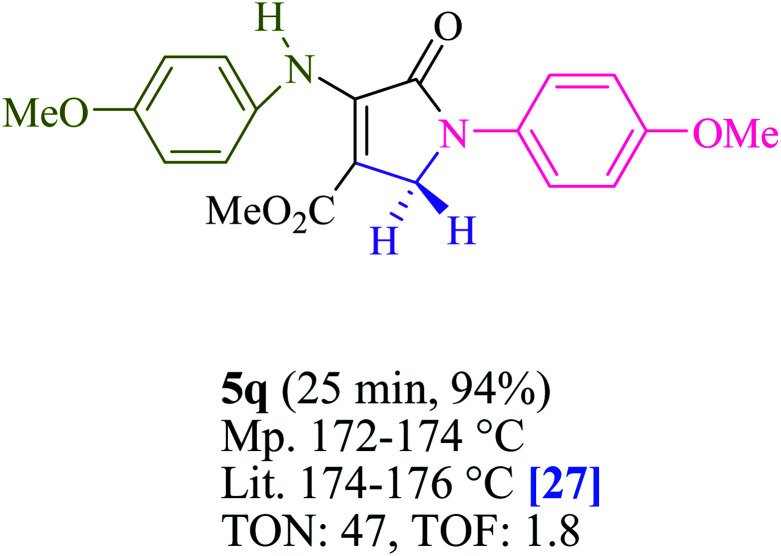	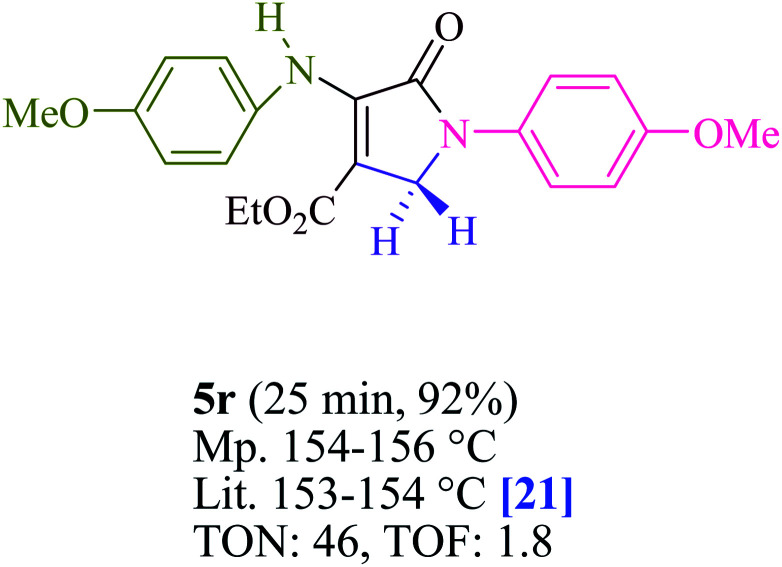
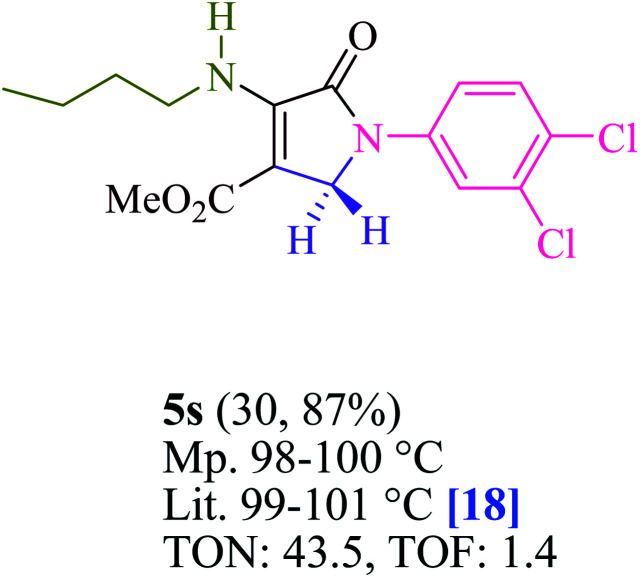	

**Scheme 2:**
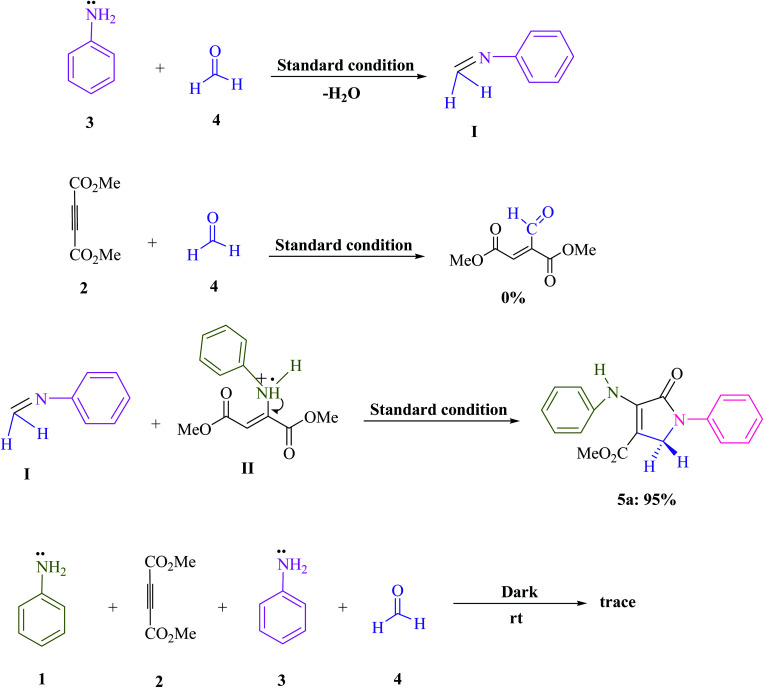
Control studies that are important for understanding the mechanism of aniline (1 and 3, 2 mmol), dimethyl acetylenedicarboxylate (DMAD) (2, 1 mmol), and formaldehyde (4, 1.5 mmol) reactions.

**Scheme 3:**
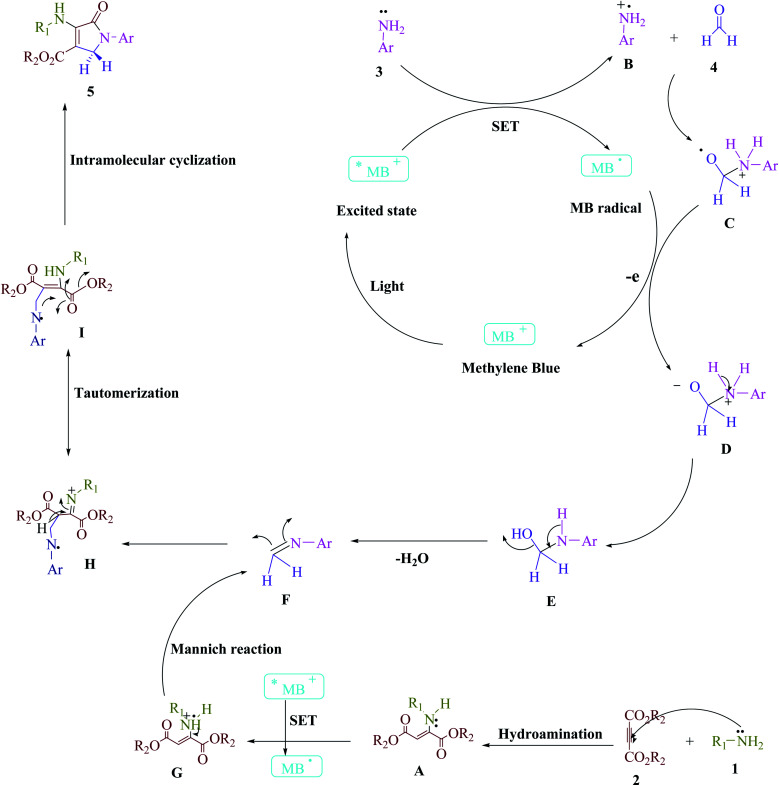
The proposed mechanistic pathway to synthesize the polyfunctionalized dihydro-2-oxypyrroles.

A series of control experiments were carried out to acquire insight into the reaction mechanism of this visible light-promoted four-component reaction. The condensation of aniline (3) with formaldehyde (4) was carried out under normal conditions (MB^+^ in EtOH under blue LED) with the removal of H_2_O to produce the equivalent imine (I), as illustrated in Scheme 2. No product was found when dimethyl acetylene dicarboxylate (DMAD) (2) was reacted with formaldehyde (4) under identical reaction conditions. However, under standard conditions, the reaction of imine (I) and enamine radical (II) produced the expected product 5a in 95% of the cases. When the reaction was carried out in the dark, a trace of the equivalent product 5a was obtained. After evaluating the results of this experiment, Scheme 3 proposes a probable reaction route in the presence of MB^+^.

Scheme 3 depicts the proposed technique. According to previous studies,^1^ this widely available cationic dye employs visible light as a source of renewable energy to construct appropriate catalytic techniques that leverage both single-electron transfer (SET) and energy transfer (EnT) pathways. More energy can be used to speed up this reaction, which can modify the visible light. The Michael reaction between amine (1) and dialkylacetylenedicarboxylate (2) produces enamine (A). The aniline radical (B) is then generated using a SET technique and visible light irradiation to increase the visible light-induced ^*^MB^+^. The radical cation (B) then interacts with formaldehyde (4) to make radical cations (C). The intermediate (D) and ground-state MB are produced as a result of the energy transfer (EnT) process between the radical adduct (C) and the MB radical (E). After that, an H_2_O molecule is extracted from (E), leaving intermediate (F). The enamine radical (G) is then generated using a SET technique to increase the visible-light-induced ^*^MB^+^. Between an activated imine (F) and an enamine radical (G), a Mannich reaction occurs, resulting in an intermediate (H) that transforms into a more stable tautomeric form (I). The intramolecular cyclization in intermediate (I) tautomerizes into comparable polyfunctionalized dihydro-2-oxypyrroles (5) in the final phase.

For the synthesis of polyfunctionalized dihydro-2-oxypyrroles, Table 3 compares the catalytic capability of a range of catalysts stated in this literature. It could have a range of applications, including the use of a small amount of photocatalyst, a quick reaction time, and the absence of by-products when using visible light irradiation. At multigram scales, the atom-economic protocol is exceedingly successful and has substantial industrial ramifications. Both in terms of efficiency and purity, these materials shine.

**Table 3:** Comparison of the catalytic ability of various catalysts for the production of polyfunctionalized dihydro-2-oxypyrroles^a^

Entry	Product	Catalyst	Conditions	Time/Yield	TON	TOF	Ref.
1	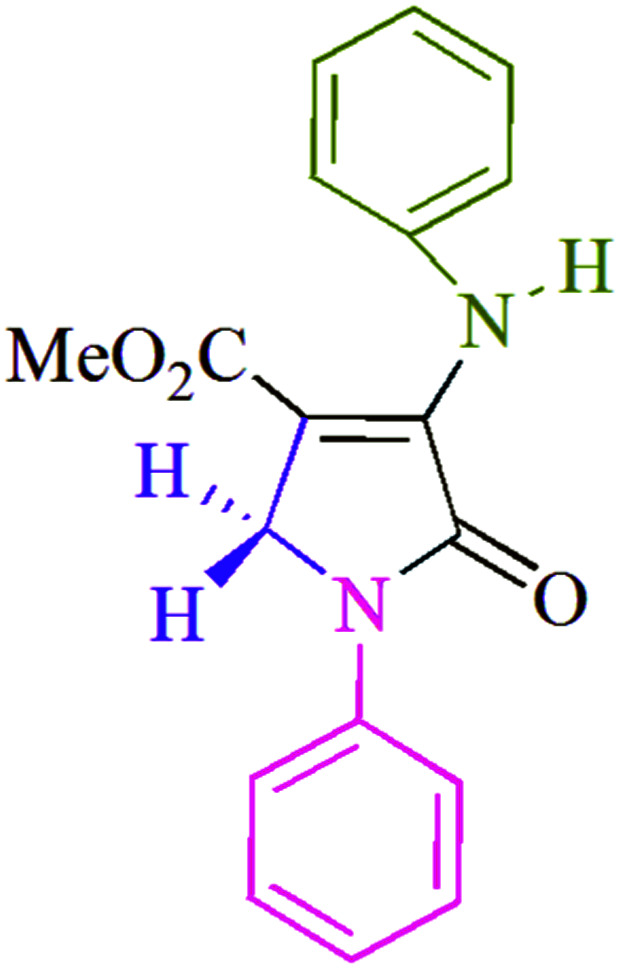	I_2_	MeOH, rt	1h/82%	8.2	0.13	17
2		Glycine	MeOH, rt	3h/93%	9.3	0.05	18
3		Glutamic acid	MeOH, rt	2h/91%	4.5	0.03	24
4		2,6-Pyridinedicarboxylic acid	MeOH, rt	1h/85%	8.5	0.14	27
**5**		**MB** ^**+**^	**Visible light irradiation, EtOH, rt**	**25 min/95%**	**47.5**	**1.9**	**This work**
6	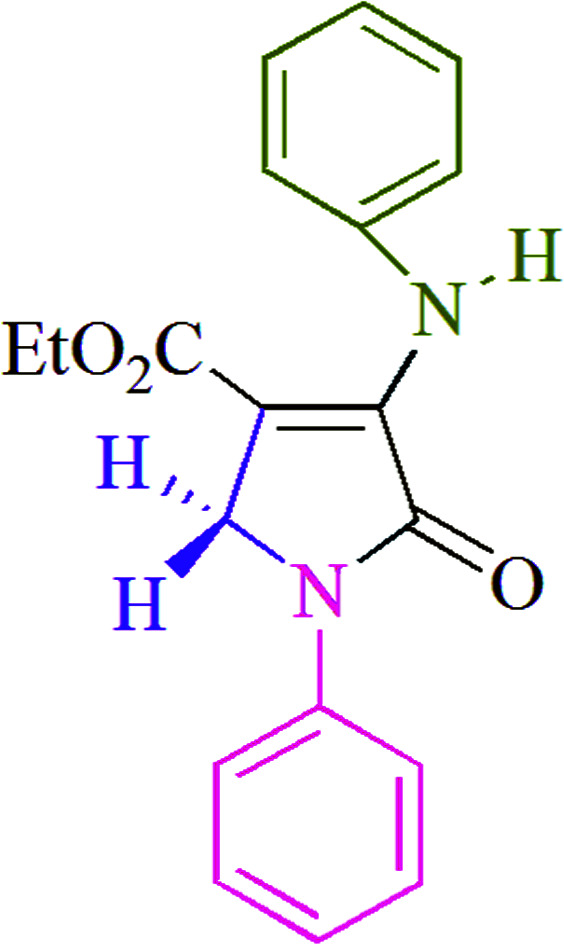	I_2_	MeOH, rt	1h/81%	8.1	0.13	17
7		Glycine	MeOH, rt	3h/90%	9	0.05	18
8		Glutamic acid	MeOH, rt	2h/88%	4.4	0.03	24
9		2,6-Pyridinedicarboxylic acid	MeOH, rt	2h/81%	8.1	0.06	27
**10**		**MB** ^**+**^	**Visible light irradiation, EtOH, rt**	**25 min/93%**	**46.5**	**1.8**	**This work**

a Aniline, dimethyl/ethylacetylenedicarboxylate, and formaldehyde are used in a four-component synthesis.

## Conclusions

According to the findings, the photo-excited state functions generated by MB^+^ can be used to metal-free manufacture polyfunctionalized dihydro-2-oxypyrroles *via* a single-electron transfer (SET)/energy transfer (EnT) method. This procedure is carried out using visible light as a renewable energy source in EtOH solvent and air atmosphere at ambient temperature. The use of the least quantity of catalysts, excellent yields, and efficient side of the reaction, secure reaction conditions, renewable energy sources, and a speedy procedure without the use of toxic solvents or catalysts are the most obvious features of this green protocol. There was no need for chromatographic purification. This reaction can be scaled up without compromising the outcome, according to a multigram scale reaction of model substrates. As a result, this process offers additional benefits in terms of meeting industrial needs and addressing environmental concerns.

## Conflicts of interest

There are no conflicts to declare.

## Supplementary Material

RA-012-D2RA01190E-s001
