# Supplementary material for: The development of imin-based tandem Michael–Mannich cyclocondensation through a single-electron transfer (SET)/energy transfer (EnT) pathway in the use of methylene blue (MB+) as a photo-redox catalyst
Source: RSC Adv. 2022 Apr 6;12(17):10701–10. doi: 10.1039/d2ra01190e (PMC8984404; doi:10.1039/d2ra01190e)
Supplement: RA-012-D2RA01190E-s001 [file RA-012-D2RA01190E-s001.pdf]

Electronic Supplementary Material (ESI) for RSC Advances.
This journal is © The Royal Society of Chemistry 2022

Supporting Information

The development of imin-based tandem Michael-Mannich cyclocondensation through a single-electron transfer (SET)/energy transfer (EnT) pathway in the use of methylene blue (MB^+) as a photo-redox catalyst

Farzaneh Mohamadpour*

School of Engineering, Apadana Institute of Higher Education, Shiraz, Iran

**Corresponding author. mohamadpour.f.7@gmail.com*

Table of content

1. TON and TOF calculation method

2. Figures

2.1. Fig. S1 The some of oxypyrrole rings are biologically active.

3. Tables

3.1. Table S1 Optimization table of photocatalyst for the synthesis of **5a**

3.2. Table S2 For the synthesis of **5a**, a table of solvent and visible-light optimization is provided

4. ^{1}HNMR, ^{13}CNMR, and mass data recorded for compounds

5. ^{1}HNMR, ^{13}CNMR, and mass files recorded for compounds

1. TON and TOF calculation method

TON=Yield/Amount of catalyst (mol), TOF=Yield/Time/Amount of catalyst (mol). The lower the amount of catalyst and the higher the yield, the higher the numerical value of the TON and TOF, and the higher the value, the more efficient the catalyst. For **5a**: TON=47.5 and TOF=1.9 and for **5b**: TON=46.5 and TOF=1.8 which is high compared to the other catalysts presented in Table 3. Given that the purpose of this study was to increase the yield, reduce the reaction rate and use the minimum amount of catalyst.

2. Figures

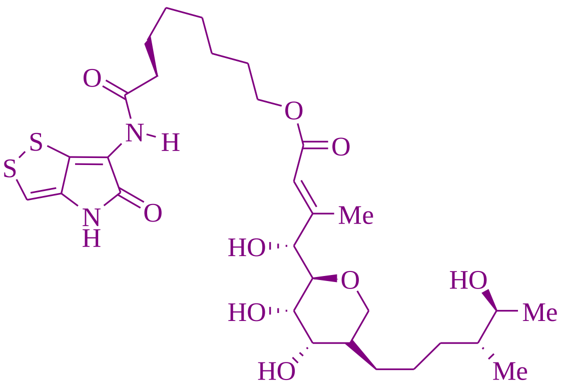

Thiomarinol A 4

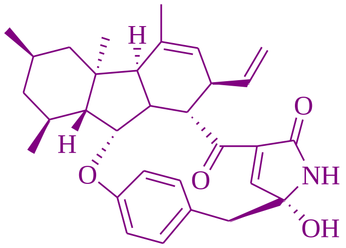

Pyrrocidine A

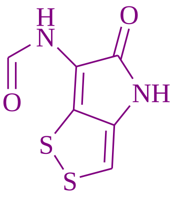

Holomycin

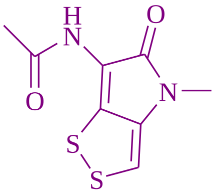

Thiolutin

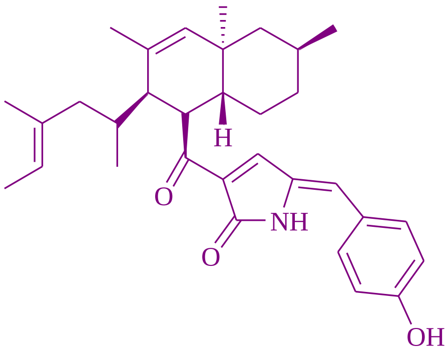

Talaroconvolutin A

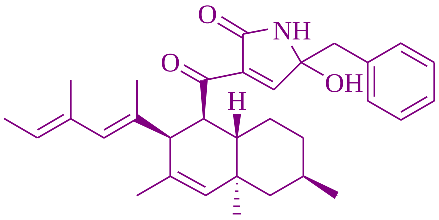

Oteromycin

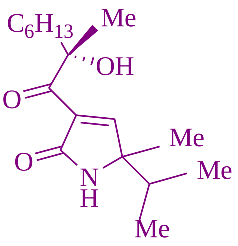

PI-o91

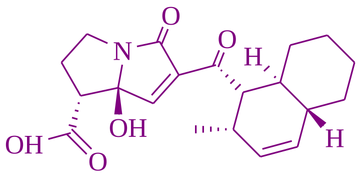

UCS1025A

Fig. S1 The some of oxypyrrole rings are biologically active.

3. Tables

Table S1 Optimization table of photocatalyst for the synthesis of **5a** [a]

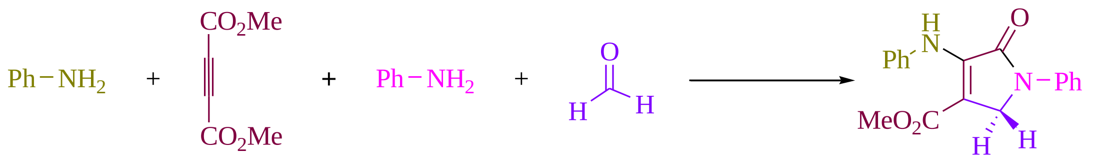

Entry	Photocatalyst	Solvent (3 mL)	Time (min)	Isolated Yields (%)
1	Methylene blue (2.5 mol%)	EtOH	25	95
2	Erythrosin B (2 mol%)	EtOH	25	71
3	Acenaphthenequinone (2 mol%)	EtOH	25	52
4	Rhodamine B (2 mol%)	EtOH	25	68
5	Alizarin (2 mol%)	EtOH	25	46
6	Riboflavin (2 mol%)	EtOH	25	65
7	Fluorescein (2 mol%)	EtOH	25	72
8	Xanthene (2 mol%)	EtOH	25	56
9	Rose bengal (2 mol%)	EtOH	25	77

10	Phenanthrenequinone (2 mol%)	EtOH	25	49
11	9*H*-Xanthen-9-one (2 mol%)	EtOH	25	58

[a]Reaction conditions: EtOH (3 mL), blue LED (18 W), and different photocatalysts at room temperature, formaldehyde (1.5 mmol), aniline (2 mmol), and dimethyl acetylenedicarboxylate (DMAD) (1 mmol).

Table S2 For the synthesis of **5a**, a table of solvent and visible-light optimization is provided[a]

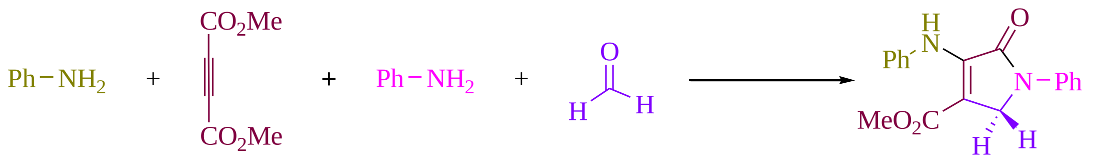

Entry	Light Source	Solvent (3 mL)	Time (min)	Isolated Yields (%)
1	Blue light (18 W)	CH_3CN	25	78
2	Blue light (18 W)	MeOH	25	84
**3**	**Blue light (18 W)**	**EtOH**	**25**	**95**
4	Blue light (18 W)	—	30	76
5	Blue light (18 W)	EtOAc	25	72
6	Blue light (18 W)	DMF	40	16
7	Blue light (18 W)	DMSO	40	23

8	Blue light (18 W)	H_2O	30	61
9	Blue light (18 W)	THF	40	13
10	Blue light (18 W)	Toluene	35	37
11	White light (18 W)	EtOH	25	90
12	Green light (18 W)	EtOH	25	82
13	__	EtOH	35	trace
14	Blue light (10 W)	EtOH	25	74
15	Blue light (12 W)	EtOH	25	82
16	Blue light (20 W)	EtOH	25	95

[a]Reaction conditions: At room temperature, formaldehyde (1.5 mmol), aniline (2 mmol), and dimethyl acetylenedicarboxylate (DMAD) (1 mmol), MB^+ (2 mol%).

4. ^{1}HNMR, ^{13}CNMR, and mass data recorded for compounds

Ethyl 1-phenyl-3-(phenylamino)-2,5-dihydro-2-oxo-1Hpyrrole-4-carboxylate (5b)

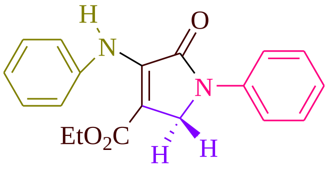

Yield: 93%; m.p. 139-141 °C; ^{1}HNMR (300 MHz, $CDCl_3$): 1.23 (3H, t, *J*= 9.6 Hz, $OCH_2\underline{CH_3}$), 4.24 (2H, q, *J*= 9.6 Hz, $O\underline{CH_2}CH_3$), 4.58 (2H, s, $\underline{CH_2}$–N), 7.16–7.25 (4H, m, ArH), 7.29-7.37 (2H, m, ArH), 7.40-7.46 (2H, m, ArH), 7.84 (2H, d, *J*= 11.6 Hz, ArH), 8.01 (1H, s, NH) ppm.

Methyl4-(4-methylphenylamino)-1-(4-methylphenyl)-2,5-dihydro-5-oxo-1H-pyrrole-3-carboxylate (5c)

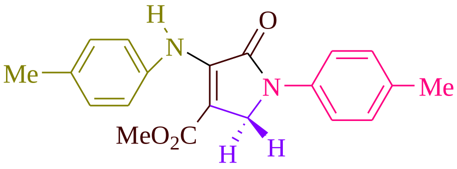

Yield: 97%; m.p. 179-181 °C; ^{1}HNMR (400 MHz, $CDCl_3$): 2.36 (6H, s, $2CH_3$), 3.77 (3H, s, OCH_3), 4.52(2H, s, $C\underline{H_2}$-N), 7.06 (2H, d, *J*=8.4 Hz, ArH), 7.14 (2H, d, *J*=8.4 Hz, ArH), 7.21(2H, d, *J*=8.4 Hz, ArH), 7.68 (2H, d, *J*=8.8 Hz, ArH), 8.03 (1H, s, NH) ppm.

Ethyl4-(4-methylphenylamino)-1-(4-methylphenyl)-2,5-dihydro-5-oxo-1H-pyrrole-3-carboxylate (5d)

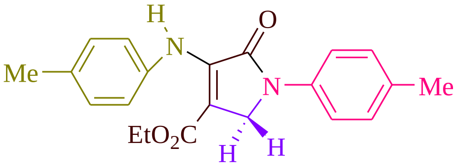

Yield: 96%; m.p. 132-134 °C; ^{1}HNMR (400 MHz, $CDCl_3$): 1.25 (3H, t, *J*=7.2 Hz, $CH_2\underline{CH_3}$), 2.37 (6H, s, $2CH_3$), 4.23 (2H, q, *J*=7.2 Hz, $2\underline{CH_2}CH_3$), 4.53 (2H, s, $C\underline{H}_2$-N),7.06 (2H, d, *J*=8.4 Hz, ArH), 7.14 (2H, d, *J*=8.4 Hz, ArH), 7.21 (2H, d, *J*=8.4 Hz, ArH), 7.68 (2H, d, *J*=8.4 Hz, ArH), 8.01 (1H, s, NH) ppm.

Methyl 3-(benzylamino)-1-(4-fluorophenyl)-2,5-dihydro-2-oxo-1H-pyrrole-4-carboxylate (5e)

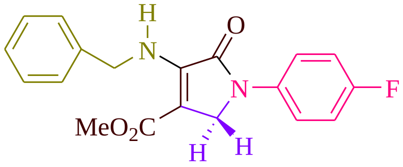

Yield: 91%; m.p. 167-169 °C; ^{1}HNMR (300 MHz, $CDCl_3$): 3.81 (s, 3H, OCH_3), 4.44 (s, 2H, $\underline{CH_2}$–N), 5.14 (d, 2H, *J*= 8.8 Hz, $\underline{CH_2}$–NH), 6.90 (br s, 1H, NH), 7.09–7.15 (m, 2H, ArH), 7.29–7.38 (m, 5H, ArH), 7.72–7.77 (m, 2H, ArH) ppm.

Methyl 3-(benzylamino)-1-(4-bromophenyl)-2,5-dihydro-2-oxo-1H-pyrrole-4-carboxylate (5f)

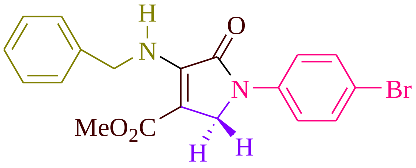

Yield: 88%; m.p. 120-122 °C; ^{1}HNMR (300 MHz, $CDCl_3$): 3.81 (3H, s, OCH_3), 4.43 (2H, s, $\underline{CH_2}$–N), 5.13 (2H, d, *J*= 8.8 Hz, $\underline{CH_2}$–NH), 6.87 (1H, br s, NH), 7.29–7.38 (5H, m, ArH), 7.51–7.55 (2H, m, ArH), 7.68–7.73 (2H, m, ArH) ppm.

Methyl4-(4-fluoroyphenylamino)-1-(4-fluorophenyl)-2,5-dihydro-5-oxo-1H-pyrrole-3-carboxylate (5g)

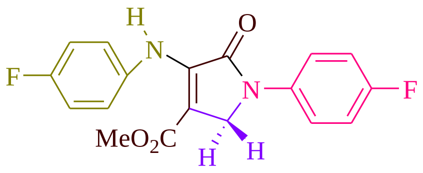

Yield: 96%; m.p. 159-161 °C; ^{1}HNMR (400 MHz, $CDCl_3$): 3.79 (3H, s, OCH_3), 4.52 (2H, s, $C\underline{H_2}$-N), 7.04 (2H, t, *J*=8.4 Hz, ArH), 7.08-7.16 (4H, m, ArH), 7.73-7.76 (2H, m, ArH), 8.05 (1H, s, NH) ppm.

Ethyl4-(4-fluoroyphenylamino)-1-(4-fluorophenyl)-2,5-dihydro-5-oxo-1H-pyrrole-3-carboxylate (5h)

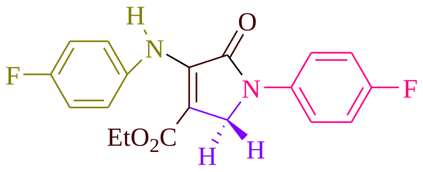

Yield: 98%; m.p. 172-174 °C; ^{1}HNMR (300 MHz, $CDCl_3$): 1.29 (3H, t, *J*= 9.2 Hz, $OCH_2\underline{CH_3}$), 4.27 (2H, q, *J*= 9.6 Hz, $O\underline{CH_2}CH_3$), 4.53 (2H, s, $\underline{CH_2}$–N), 7.01-7.17 (5H, m, ArH), 7.72–7.79 (2H, m, ArH), 8.05 (1H, s, NH) ppm.

Methyl4-(4-bromophenylamino)-1-(4-bromophenyl)-2,5-dihydro-5-oxo-1H-pyrrole-3-carboxylate (5i)

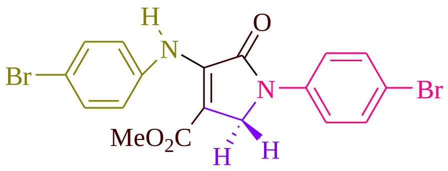

Yield: 83%; m.p. 180-182 °C; ^{1}HNMR (300 MHz, $CDCl_3$): 3.81 (3H, s, OCH_3), 4.52 (2H, s, $\underline{CH_2}$–N), 7.04 (2H, d, *J*= 11.2 Hz, ArH), 7.46 (2H, d, *J*= 11.6 Hz, ArH), 7.53 (2H, d, *J*= 12.0 Hz, ArH), 7.71 (2H, d, *J*= 12.0 Hz, ArH), 8.06 (1H, s, NH) ppm.

Ethyl3-(4-bromophenylamino)-1-(4-bromophenyl)-2,5-dihydro-2-oxo-1H-pyrrole-4-carboxylate (5j)

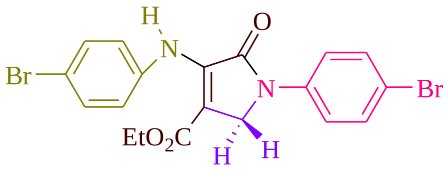

Yield: 86%; m.p. 167-169 °C; ^{1}HNMR (300 MHz, $CDCl_3$): 1.29 (3H, t, *J*= 9.6 Hz, $OCH_2\underline{CH_3}$), 4.28 (2H, q, *J*= 9.6 Hz, $O\underline{CH_2}CH_3$), 4.53 (2H, s, $\underline{CH_2}$–N), 7.04 (2H, d, *J*= 11.6 Hz, ArH), 7.45 (2H, d, *J*= 11.2 Hz, ArH), 7.53 (2H, d, *J*= 12.0 Hz, ArH), 7.72 (2H, d, *J*= 11.6 Hz, ArH), 8.05 (1H, s, NH) ppm.

Methyl4-(4-ethylphenylamino)-1-(4-ethylphenyl)-2,5-dihydro-5-oxo-1H-pyrrole-3-carboxylate (5k)

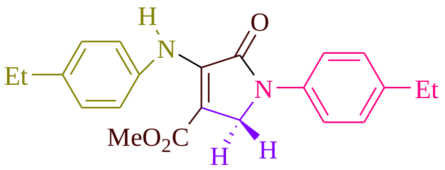

Yield: 91%; m.p. 122-124 °C; ^{1}HNMR (400 MHz, $CDCl_3$): 1.26 (6H, t, *J*=2.4 Hz, $2CH_2\underline{CH_3}$), 2.67 (4H, q, *J*=7.2 Hz, $2\underline{CH_2}CH_3$), 3.76 (3H, s, $2OCH_3$), 4.53 (2H, s, $C\underline{H}_2$-N),7.09 (2H, d, *J*=8.4 Hz, ArH), 7.17 (2H, d, *J*=8.4 Hz, ArH), 7.24 (2H, d, *J*=8.8 Hz, ArH),7.70 (2H, d, *J*=8.8 Hz, ArH), 8.05 (1H, s, NH) ppm; ^{13}CNMR (100 MHz, $CDCl_3$): 15.6, 15.7 ($2CH_2$-$\underline{CH_3}$), 28.3 and 28.4 ($2\underline{CH_2}$-CH_3), 48.3 (CH_2N), 51.3 (OCH_3), 101.9, 119.4, 123.1, 127.8, 128.5, 136.1, 136.4, 140.8, 141.3, 143.6 (C_{Ar}), 163.6 (C=O, amide), 165.1(C=O, ester); MS (EI) m/z (%): 364 (M , 59), 349 (1), 332 (10), 318 (4), 305 (100), 290 (1), 277 (7), 261 (4), 247 (2), 233 (2), 216 (13), 199 (2), 186 (1), 173 (14), 158 (12), 145 (3), 132 (18), 118 (10), 103 (12), 90 (8), 77 (21), 64 (2), 51 (4).

Ethyl4-(4-ethylphenylamino)-1-(4-ethylphenyl)-2,5-dihydro-5-oxo-1H-pyrrole-3-carboxylate (5l)

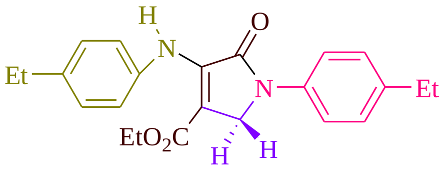

Yield: 93%; m.p. 101-103 °C; ^{1}HNMR (400 MHz, $CDCl_3$): 1.24 (9H, m, 3 $CH_2\underline{CH_3}$), 2.67 (4H, q, *J*=7.2 Hz, $2\underline{CH_2}CH_3$), 4.22 (2H, q, *J*=7.2 Hz, $\underline{CH_2}CH_3$), 4.54 (2H, s, $C\underline{H}_2$-N), 7.09 (2H, d, *J*=8.4 Hz, ArH), 7.16 (2H, d, *J*=8.4 Hz, ArH), 7.24 (2H, d, *J*=8.4 Hz, ArH), 7.71 (2H, d, *J*=8.8 Hz, ArH), 8.01 (1H, s, NH) ppm; ^{13}CNMR (100 MHz, $CDCl_3$): 14.2, 15.6 and 15.7 ($3CH_2$-$\underline{CH_3}$), 28.3 and 28.4 ($2\underline{CH_2}$-CH_3), 48.4 (CH_2N), 60.3 ($O\underline{CH_2}$-CH_3), 102.5, 119.5, 122.8, 127.8, 128.7,

136.3, 136.4, 140.7, 141.3 and 143.1 (C_{Ar}), 163.6 (C=O, amide), 164.8 (C=O, ester); MS (EI) m/z (%): 378 (M , 2), 357 (2), 339 (3), 321 (4), 305 (5), 292 (7), 275 (4), 262 (7), 239 (12), 218 (5), 199 (11), 185 (5), 171 (8), 152 (10), 130 (25), 105 (35), 91 (93), 77 (57), 57 (56), 43 (100).

Methyl 3-(benzylamino)-1-phenyl-2,5-dihydro-2-oxo-1H-pyrrole-4-carboxylate (5m)

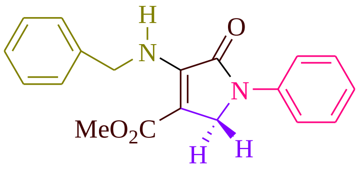

Yield: 92%; m.p. 138-140 °C; ^{1}HNMR (300 MHz, $CDCl_3$): 3.81 (3H, s, OCH_3), 4.47 (2H, s, $\underline{CH_2}$–N), 5.15 (2H, d, *J*= 8.8 Hz, $\underline{CH_2}$–NH), 6.86 (1H, br, NH), 7.20–7.47 (8H, m, ArH), 7.79 (2H, d, *J*= 10.0 Hz, ArH) ppm.

Ethyl 3-(benzylamino)-1-phenyl-2,5-dihydro-2-oxo-1Hpyrrole-4-carboxylate (5n)

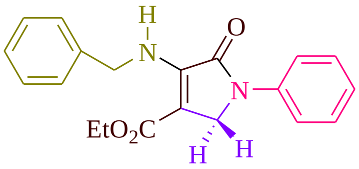

Yield: 89%; m.p. 131-133 °C; ^{1}HNMR (300 MHz, $CDCl_3$): 1.35 (3H, t, *J*= 9.6 Hz, $OCH_2\underline{CH_3}$), 4.28 (2H, q, *J*= 9.6 Hz, $O\underline{CH_2}CH_3$), 4.47 (2H, s, $\underline{CH_2}$–N), 5.15 (2H, d, *J*= 8.8 Hz, $\underline{CH_2}$–NH), 6.85 (1H, br, NH), 7.25–7.46 (8H, m, ArH), 7.80 (2H, d, *J*= 9.6 Hz, ArH) ppm.

Methyl 3-(butylamino)-2,5-dihydro-2-oxo-1-phenyl-1Hpyrrole-4-carboxylate (5o)

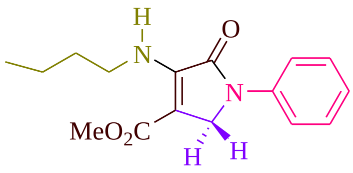

Yield: 95%; m.p. 57-59 °C; ^{1}HNMR (300 MHz, $CDCl_3$): 0.98 (3H, t, *J*= 9.6 Hz, CH_3), 1.42 (2H, sextet, *J*= 9.2 Hz, CH_2), 1.64 (2H, quintet, *J*= 9.0 Hz, CH_2), 3.82 (3H, s, OCH_3), 3.89 (2H, t, *J*= 9.2 Hz, $\underline{CH_2}$–NH), 4.44 (2H, s, $\underline{CH_2}$–N), 6.67 (1H, br s, NH), 7.19-7.24 (1H, m, ArH), 7.40-7.45 (2H, m, ArH), 7.79 (2H, d, *J*= 10.4 Hz, ArH) ppm.

Ethyl 1-(4-bromophenyl)-3-(butylamino)-2,5-dihydro-2-oxo-1H-pyrrole-4-carboxylate (5p)

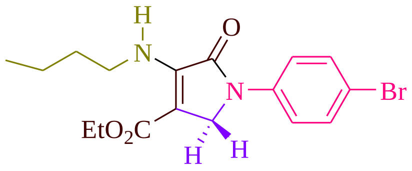

Yield: 86%; m.p. 97-99 °C; ^{1}HNMR (400 MHz, $CDCl_3$): 0.97 (3H, t, *J* = 7.2 Hz, CH_3), 1.35 (3H, t, *J* = 7.2 Hz, $OCH_2\underline{CH_3}$), 1.43 (2H, sextet, *J* = 7.6 Hz, CH_2), 1.61 (2H, quintet, *J* = 7.6 Hz, CH_2), 3.87 (2H, t, *J* = 7.2 Hz, $\underline{CH_2}$-NH), 4.28 (2H, q, *J* = 7.2 Hz, $O\underline{CH_2}CH_3$), 4.40 (2H, s, $\underline{CH_2}$-N), 6.72 (1H, br s, NH), 7.52 (2H, d, *J* = 8.8 Hz, ArH), 7.70 (2H, d, *J* = 8.8 Hz, ArH) ppm.

Methyl4-(4-methoxyphenylamino)-1-(4-methoxyphenyl)-2,5-dihydro-5-oxo-1H-pyrrole-3-carboxylate (5q)

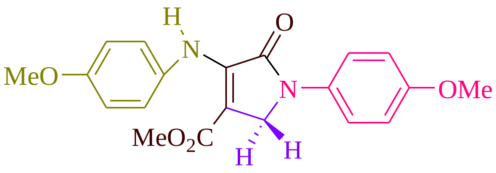

Yield: 94%; m.p. 172-174 °C; ^{1}HNMR (400 MHz, $CDCl_3$): 3.77 (3H, s, CH_3), 3.83 (6H, s, $2OCH_3$), 4.50 (2H, s, C<u>H</u>$_2$-N), 6.89 (4H, d, *J*=17.6 Hz, ArH), 7.13 (1H, s, ArH) ,7.68 (1H, s, ArH), 8.03 (1H, s, NH) ppm.

Ethyl4-(4-methoxyphenylamino)-1-(4-methoxyphenyl)-2,5-dihydro-5-oxo-1H-pyrrole-3-carboxylate (5r)

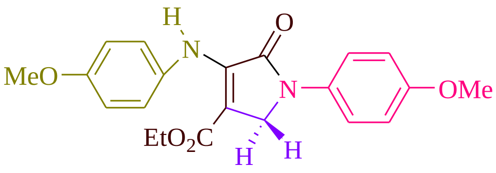

Yield: 92%; m.p. 154-156 °C; ^{1}HNMR (400 MHz, $CDCl_3$): 1.26 (3H, t, *J*=7.2Hz, $CH_2\underline{CH_3}$), 3.83 (6H, s, $2OCH_3$), 4.23 (2H, q, *J*=7.2 Hz, $\underline{CH_2}CH_3$), 4.50 (2H, s, C<u>H</u>$_2$-N), 6.87 (2H, d, *J*=8.8 Hz, ArH), 6.93 (2H, d, *J*=8.8 Hz, ArH), 7.12 (2H, d, *J*=8.8 Hz, ArH), 7.69 (2H, d, *J*=8.8 Hz, ArH), 8.02 (1H, s, NH) ppm.

Methyl1-(3,4-di-chlorophenyl)-3-(butylamino)-2,5-dihydro-2-oxo-1H-pyrrole-4-carboxylate (5s)

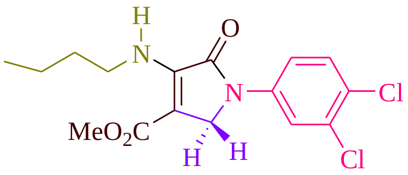

Yield: 87%; m.p. 98-100 °C; ^{1}HNMR (300 MHz, $CDCl_3$): 0.98 (3H, t, *J*= 9.6 Hz, CH_3), 1.46 (2H, sextet, *J*= 10.4 Hz, CH_2), 1.62 (2H, quintet, *J*= 10.0 Hz, CH_2), 3.82 (3H, s, OCH_3), 3.89 (2H, t, *J*= 9.2 Hz, $\underline{CH_2}$–NH), 4.40 (2H, s, $\underline{CH_2}$–N), 6.69 (1H, br s, NH), 7.45-7.48 (1H, m, ArH), 7.66-7.70 (1H, m, ArH), 8.01 (1H, s, ArH) ppm.

5. ^{1}HNMR, ^{13}CNMR, and mass files recorded for compounds

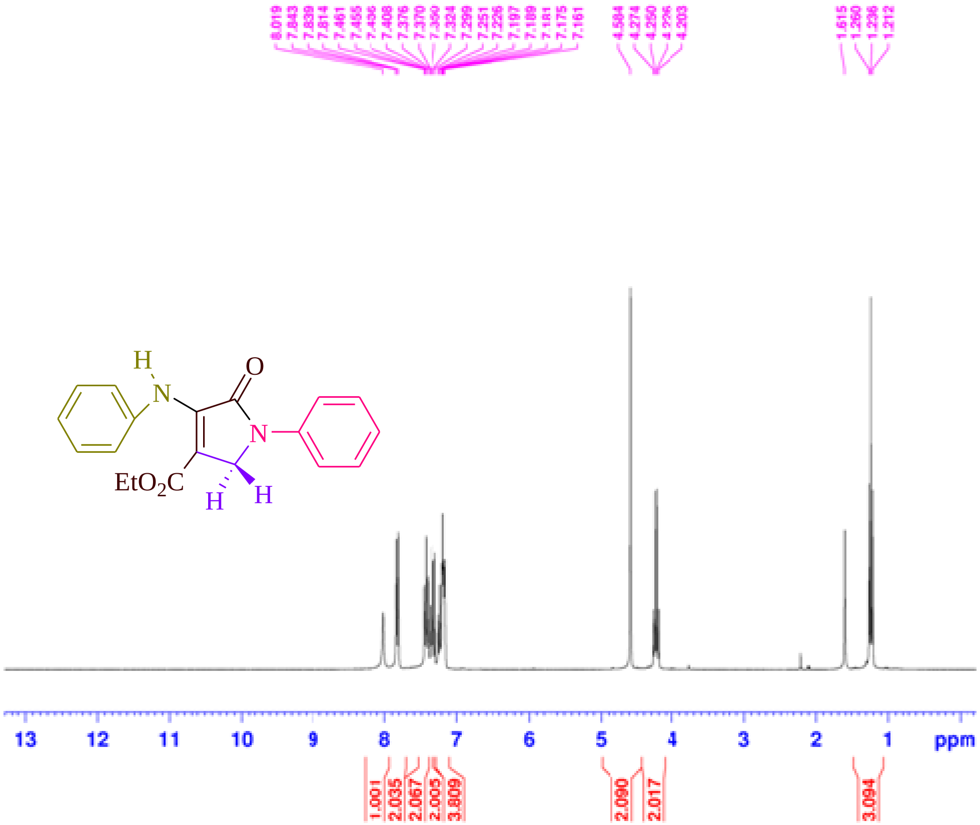

Fig. S2 ^{1}HNMR Spectrum of compound (300 MHz, $CDCl_3$) of **5b**

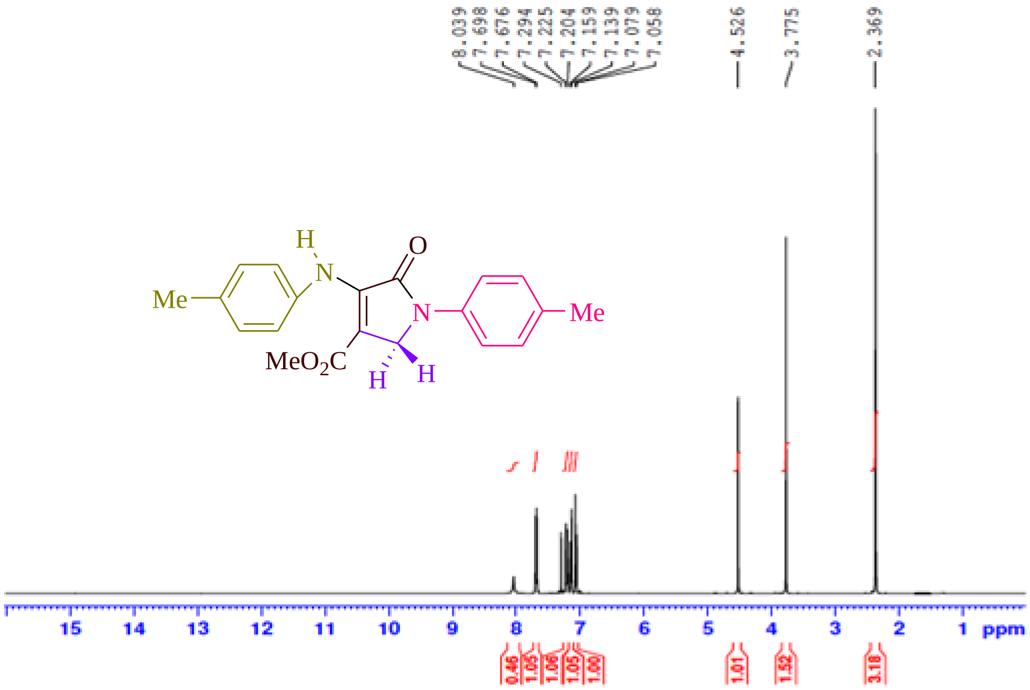

Fig. S3 ^{1}HNMR Spectrum of compound (400 MHz, $CDCl_3$) of **5c**

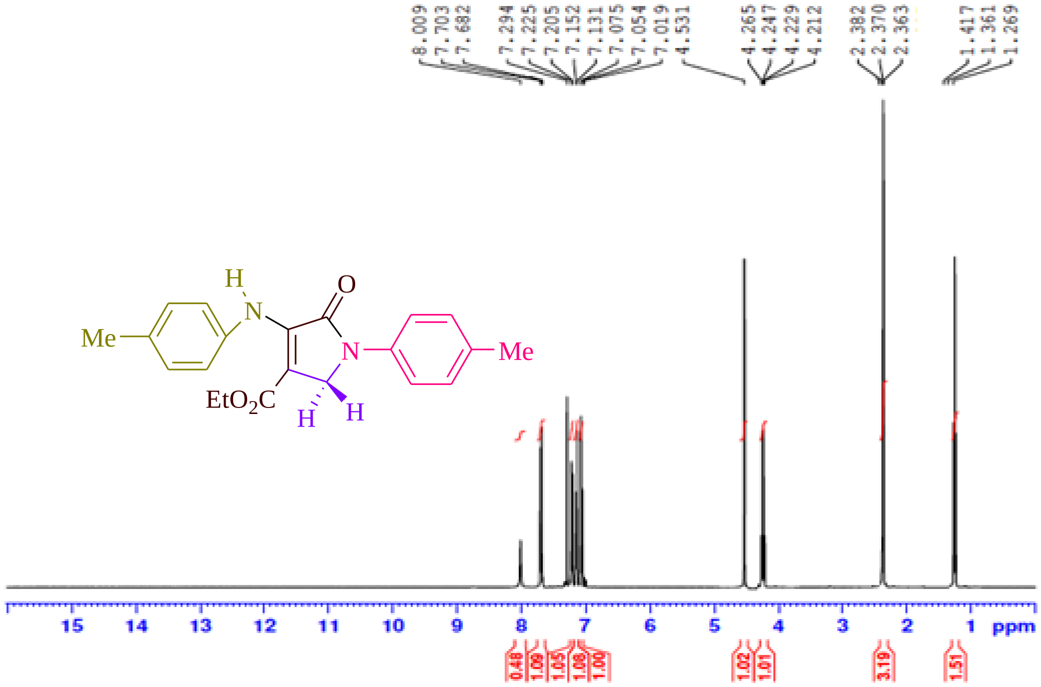

Fig. S4 ^{1}HNMR Spectrum of compound (400 MHz, $CDCl_3$) of **5d**

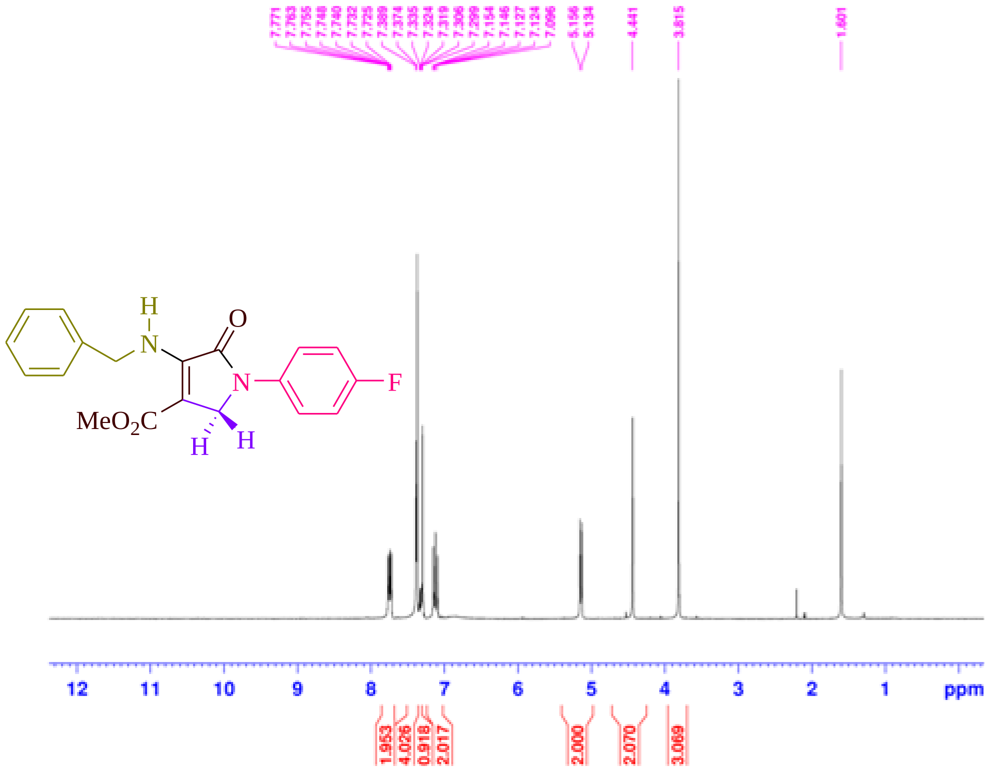

Fig. S5 ^{1}HNMR Spectrum of compound (300 MHz, $CDCl_3$) of **5e**

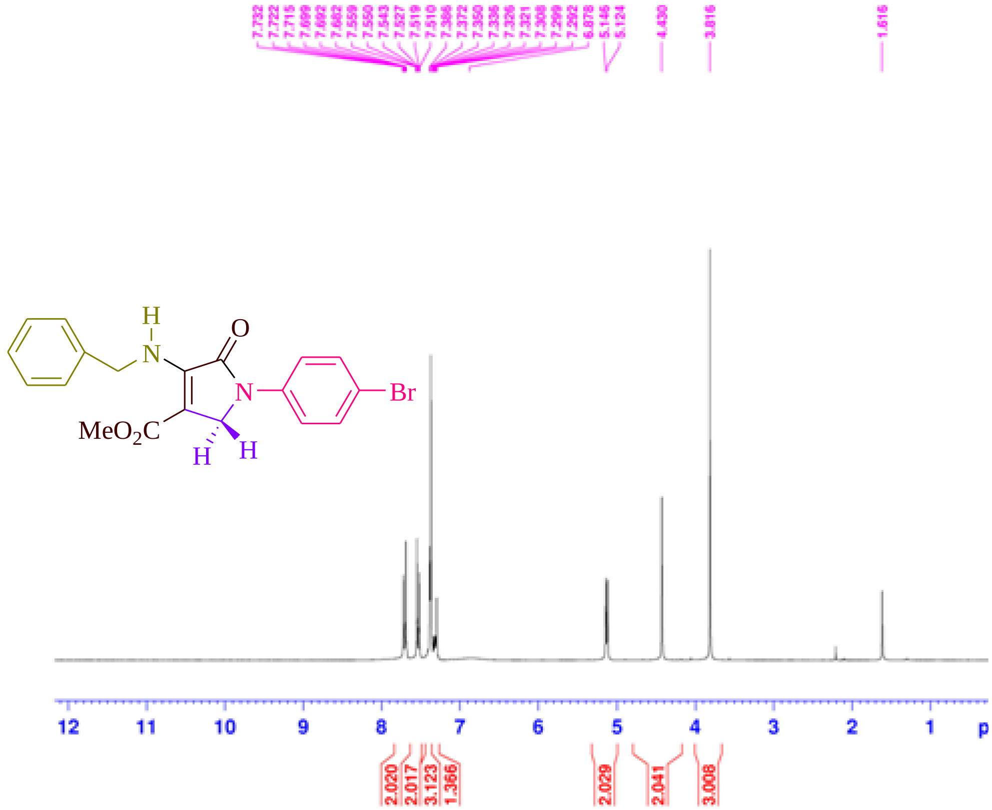

Fig. S6 ^{1}HNMR Spectrum of compound (300 MHz, $CDCl_3$) of **5f**

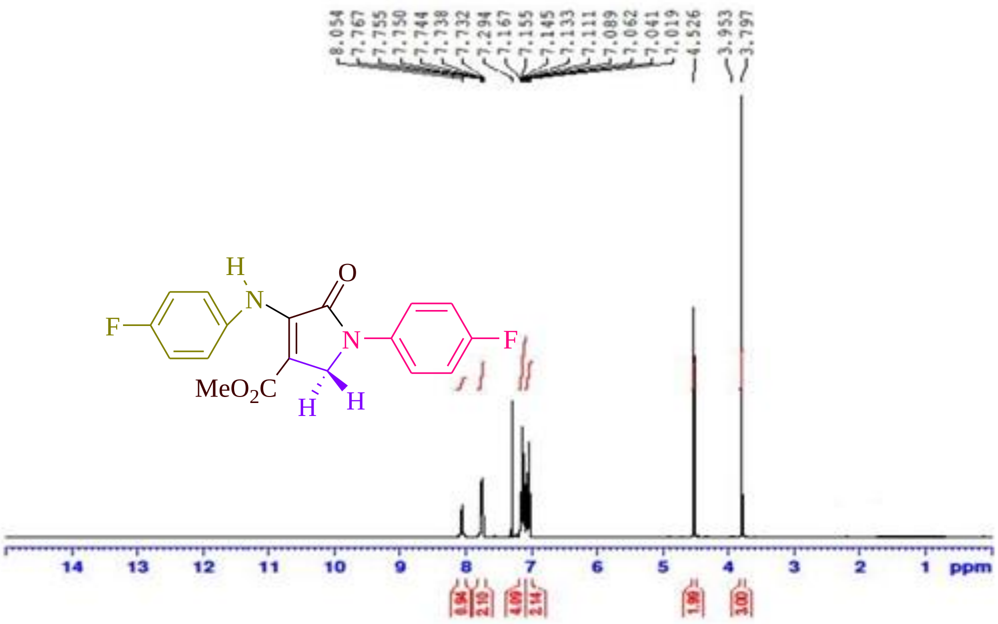

Fig. S7 ^{1}HNMR Spectrum of compound (400 MHz, $CDCl_3$) of **5g**

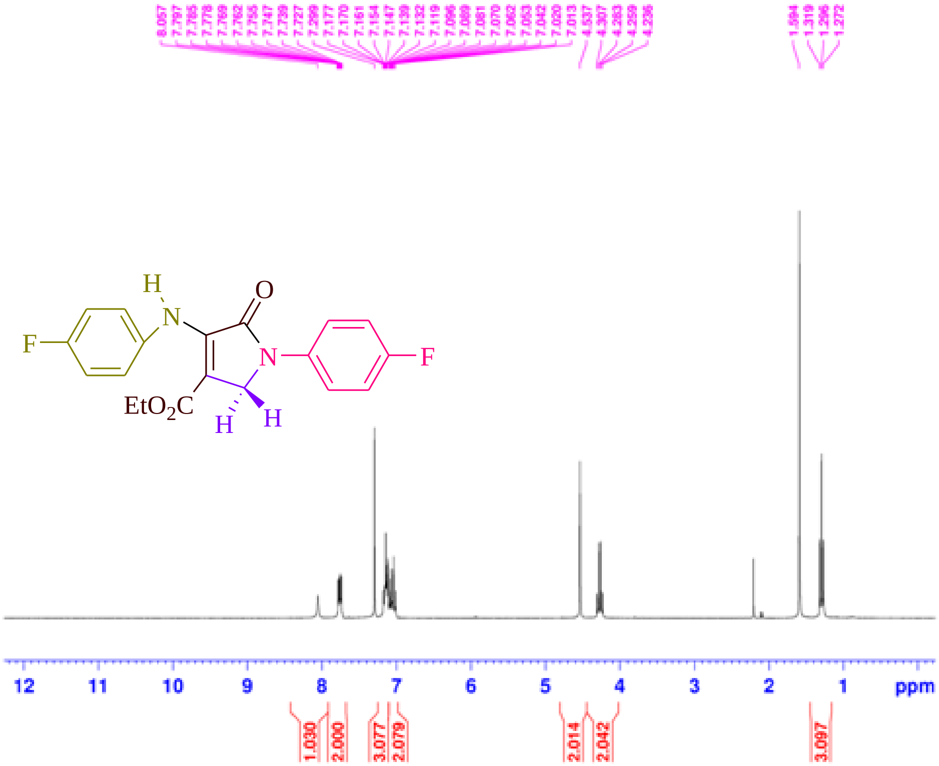

Fig. S8 ^{1}HNMR Spectrum of compound (300 MHz, $CDCl_3$) of **5h**

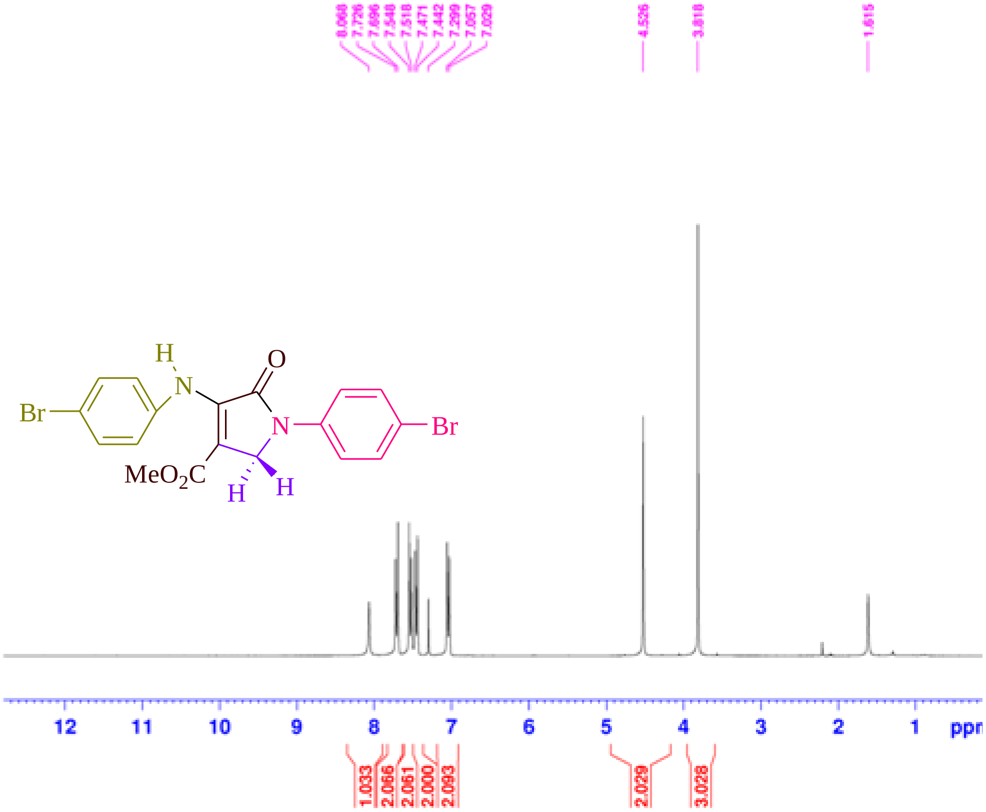

Fig. S9 ^{1}HNMR Spectrum of compound (300 MHz, $CDCl_3$) of **5i**

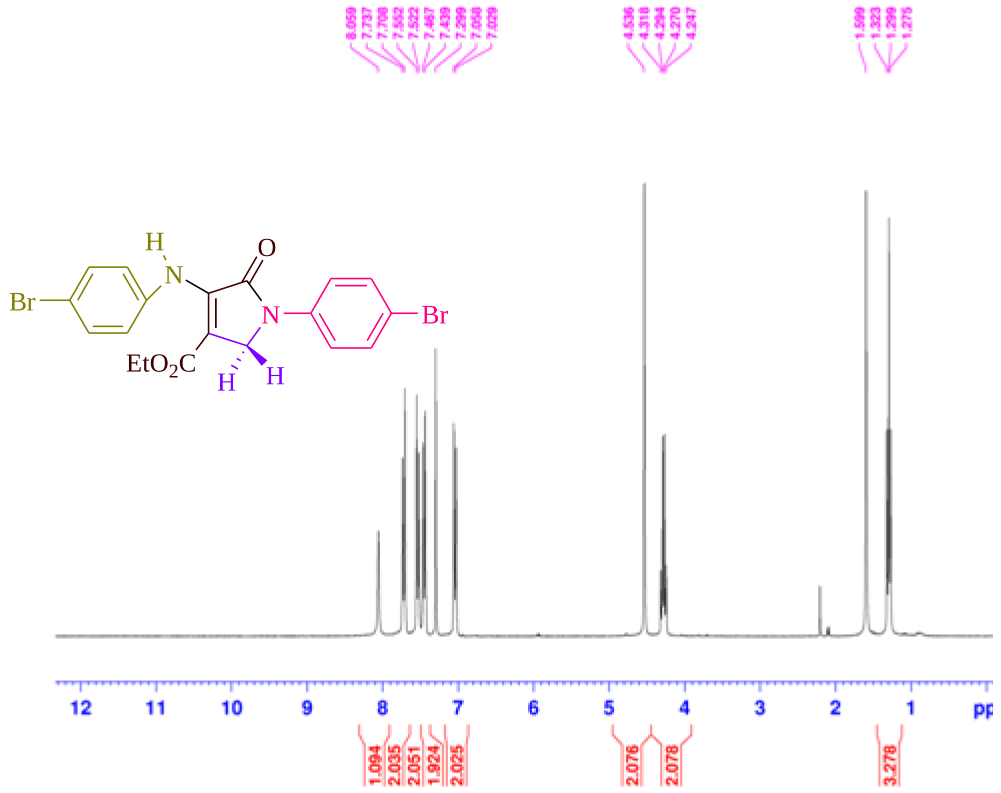

Fig. S10 ^{1}HNMR Spectrum of compound (300 MHz, $CDCl_3$) of **5j**

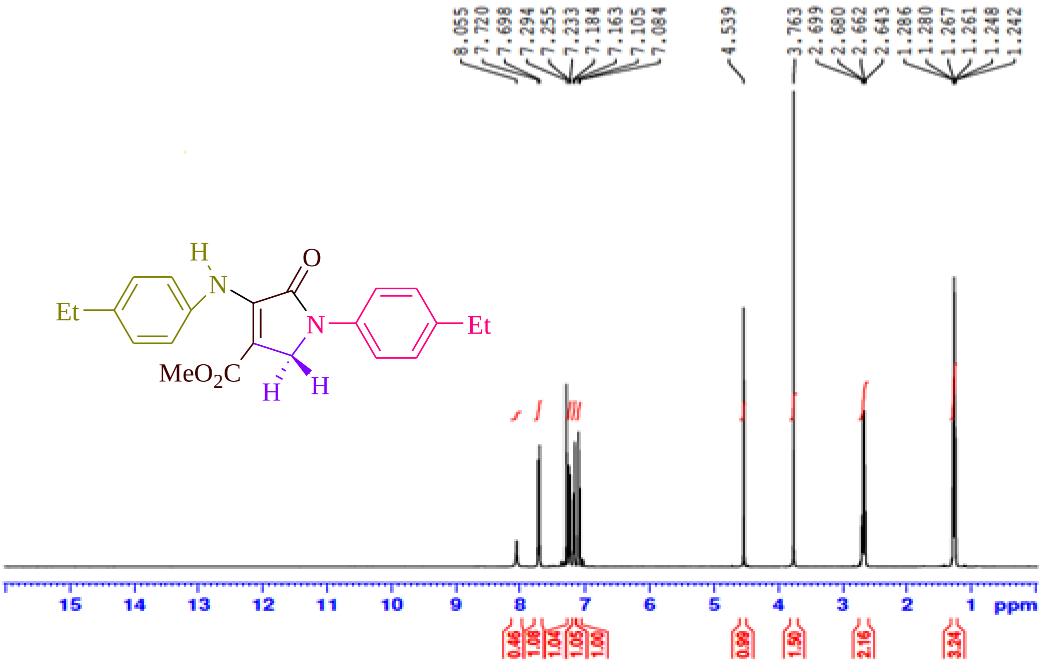

Fig. S11 ^{1}HNMR Spectrum of compound (400 MHz, $CDCl_3$) of **5k**

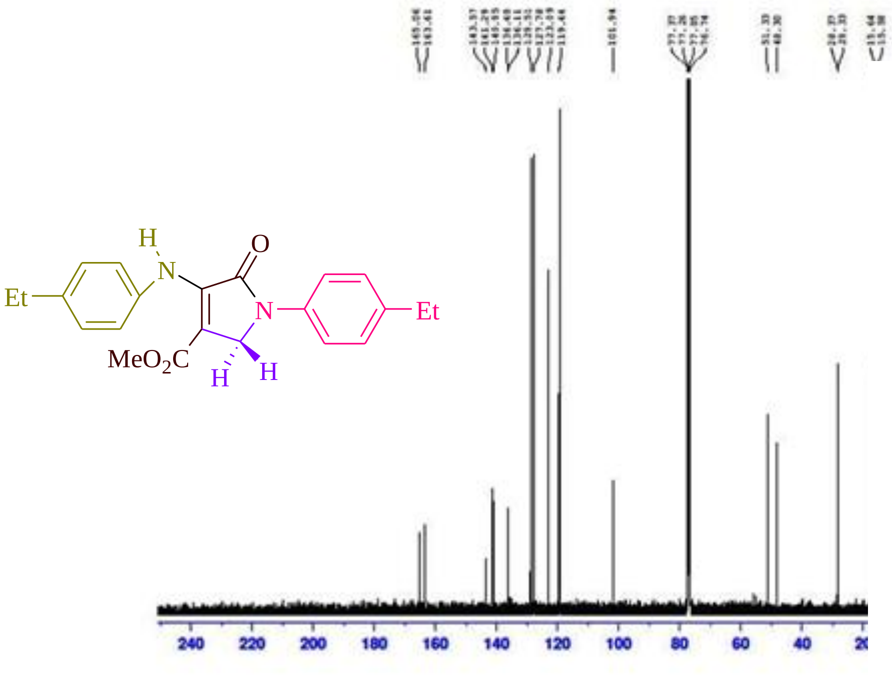

Fig. S12 ^{13}CNMR Spectrum of compound (100 MHz, $CDCl_3$) of **5k**

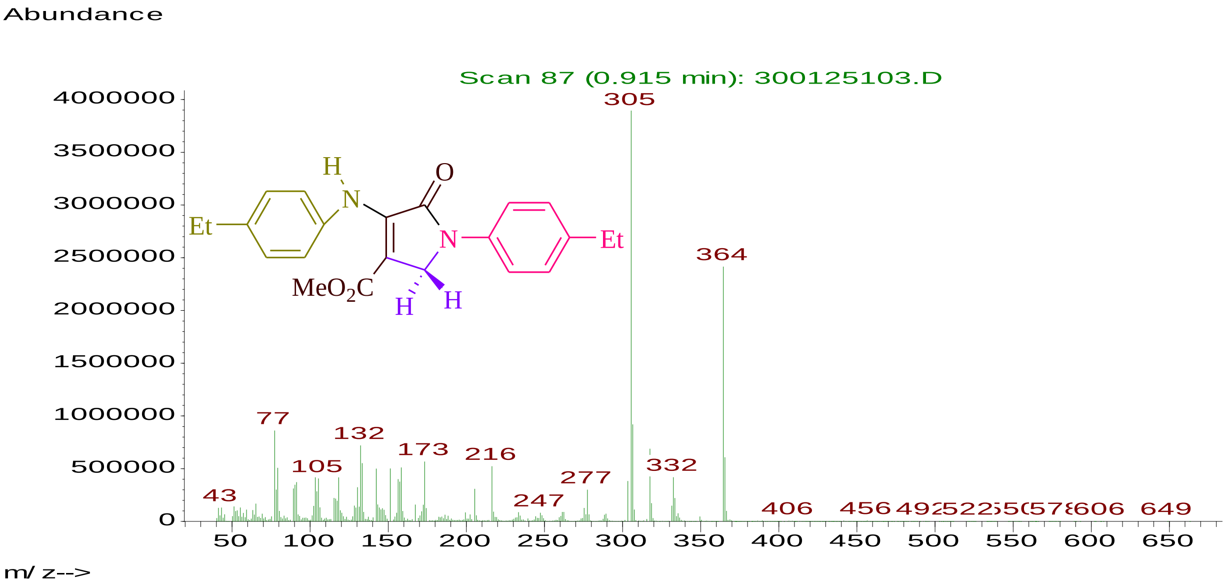

Fig. S13 Mass spectrum of compound **5k**

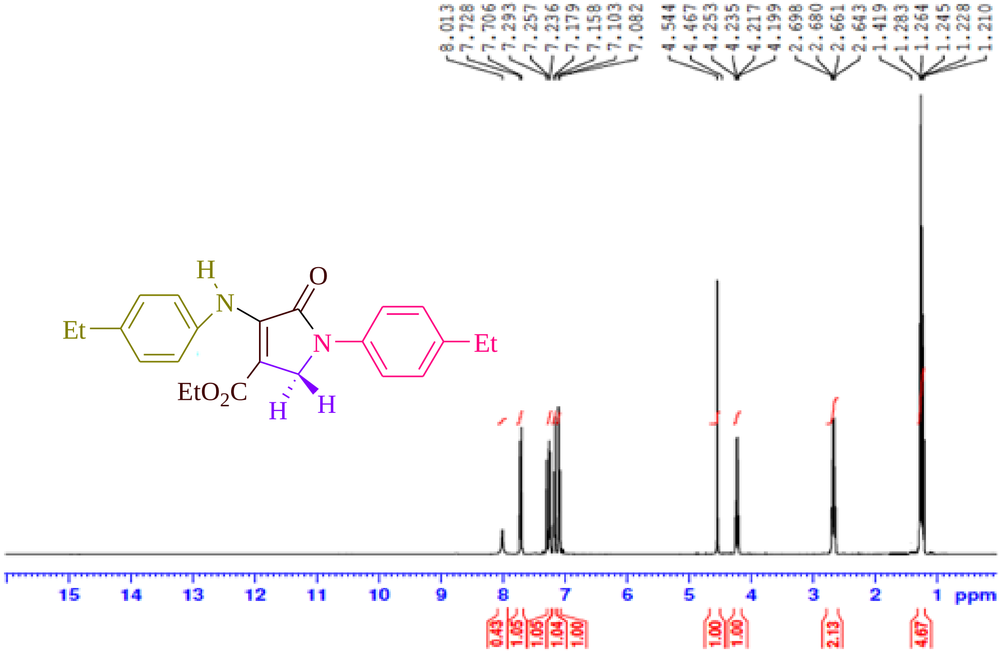

Fig. S14 ^{1}HNMR Spectrum of compound (400 MHz, $CDCl_3$) of **5l**

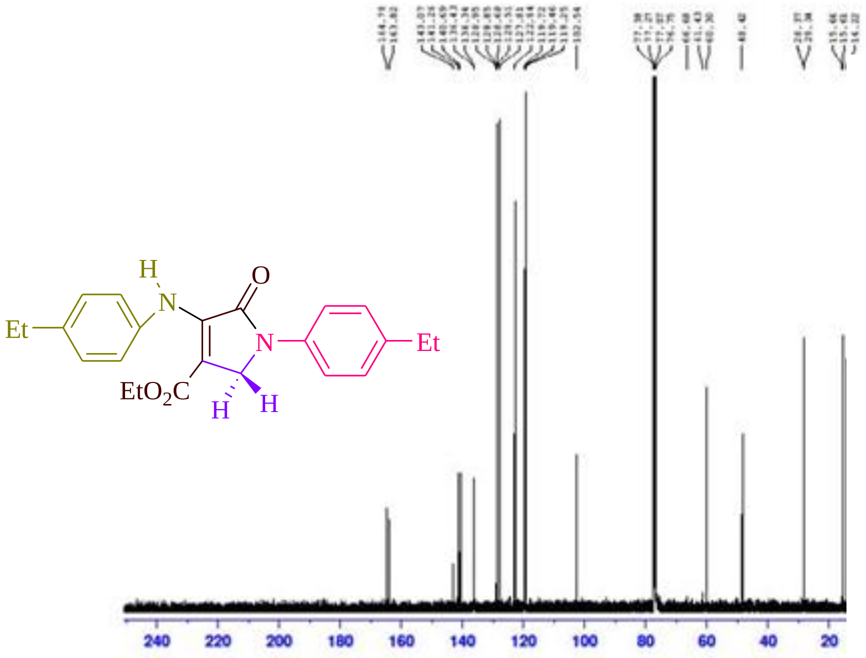

Fig. S15 ^{13}CNMR Spectrum of compound (100 MHz, $CDCl_3$) of **5l**

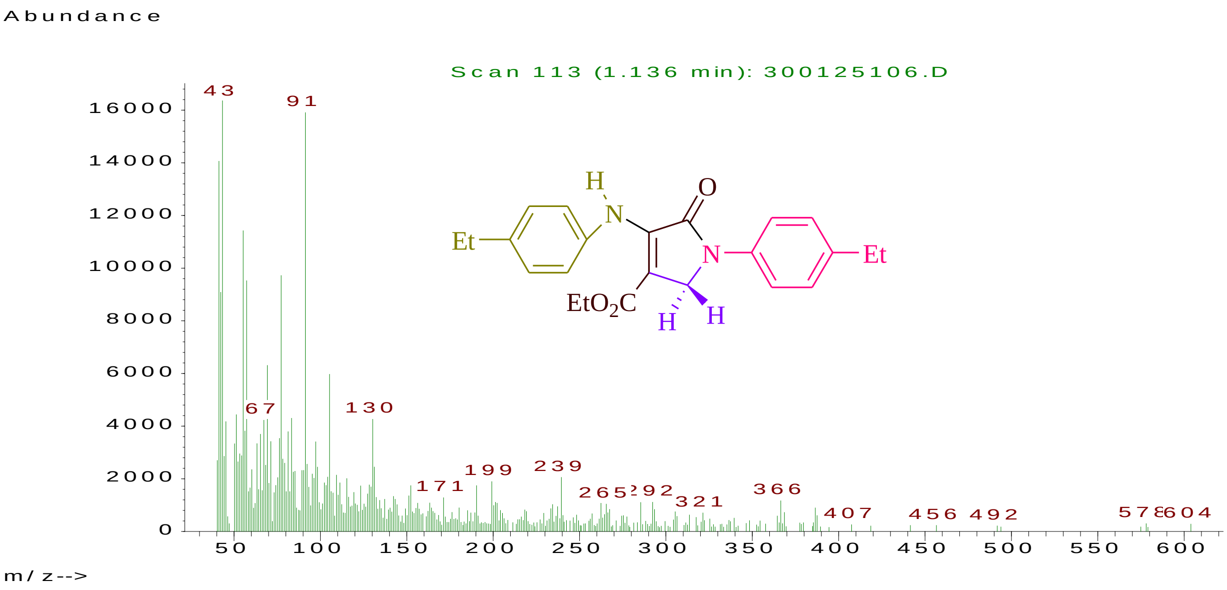

Fig. S16 Mass spectrum of compound **5l**

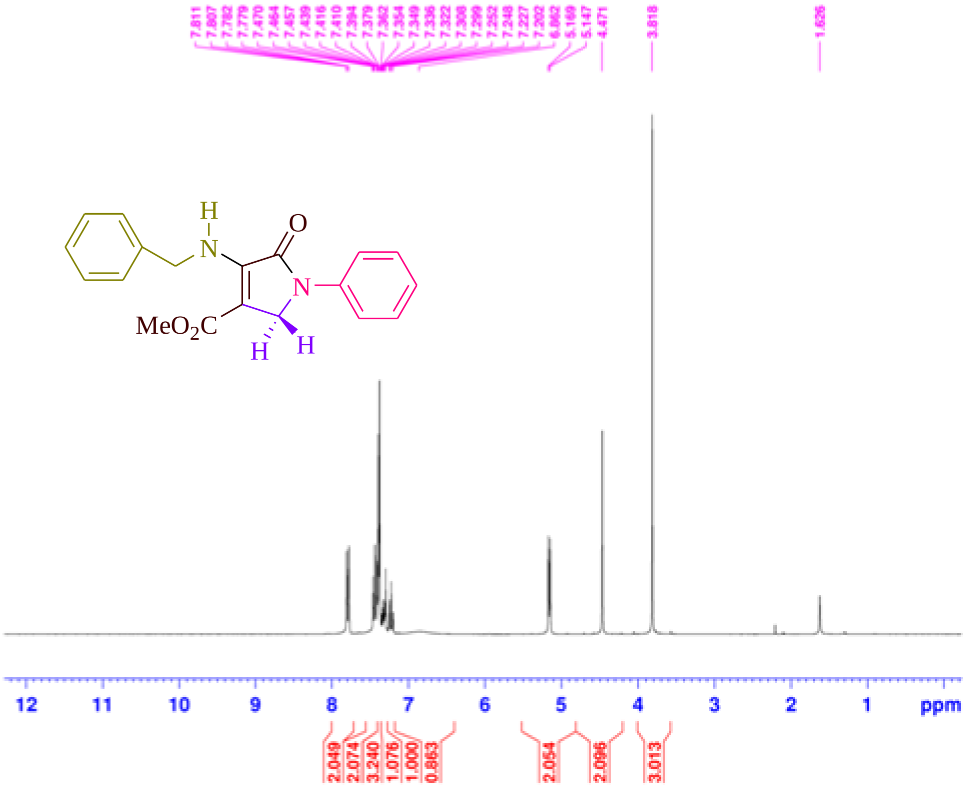

Fig. S17 ^{1}HNMR Spectrum of compound (300 MHz, $CDCl_3$) of **5m**

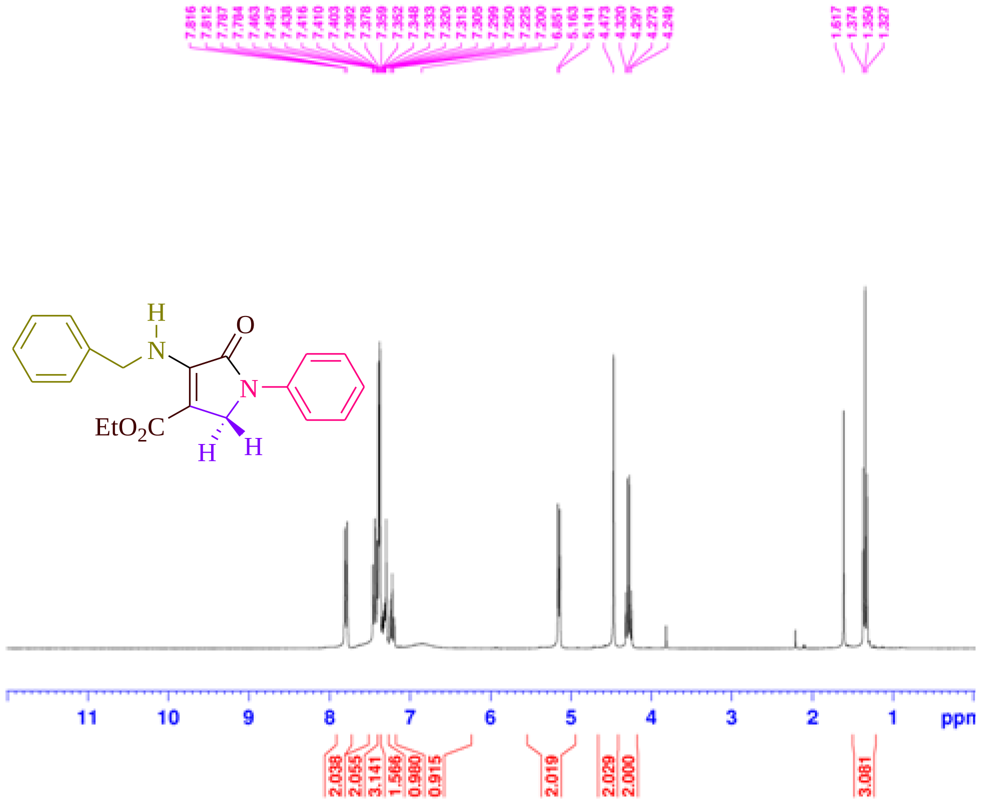

Fig. S18 ^{1}HNMR Spectrum of compound (300 MHz, $CDCl_3$) of **5n**

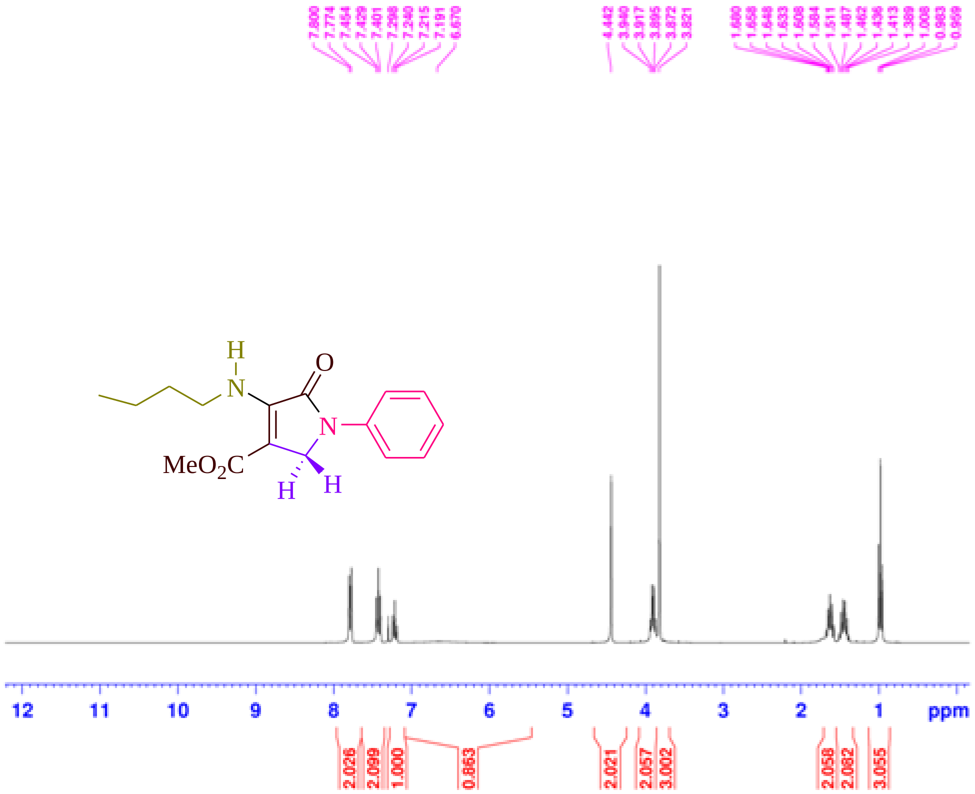

Fig. S19 ^{1}HNMR Spectrum of compound (300 MHz, $CDCl_3$) of **5o**

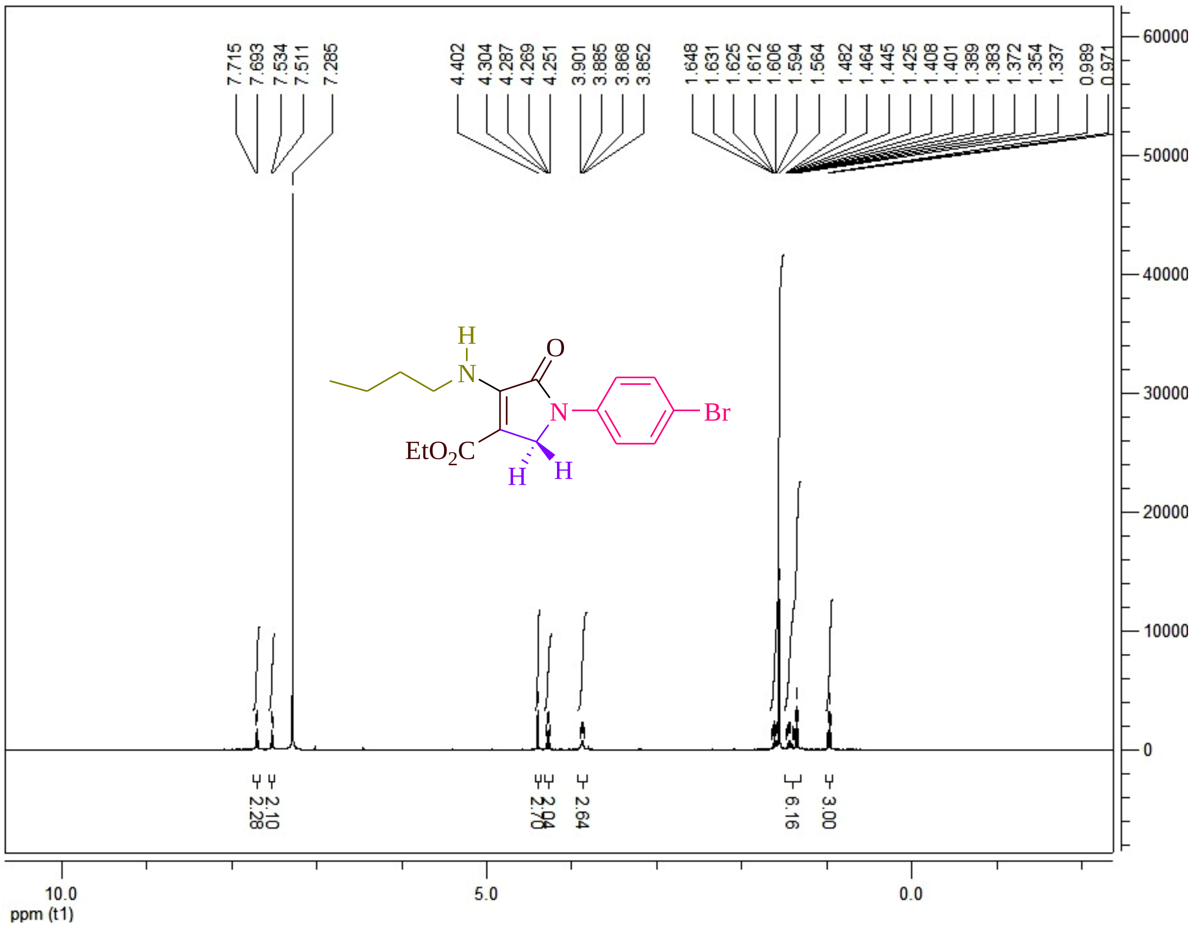

Fig. S20 ^{1}HNMR Spectrum of compound (400 MHz, $CDCl_3$) of **5p**

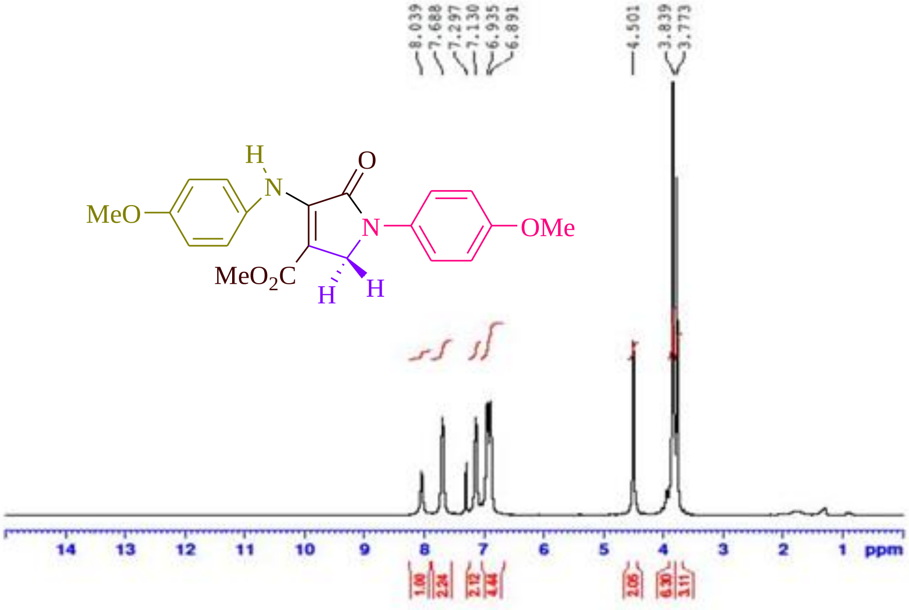

Fig. S21 ^{1}HNMR Spectrum of compound (400 MHz, $CDCl_3$) of **5q**

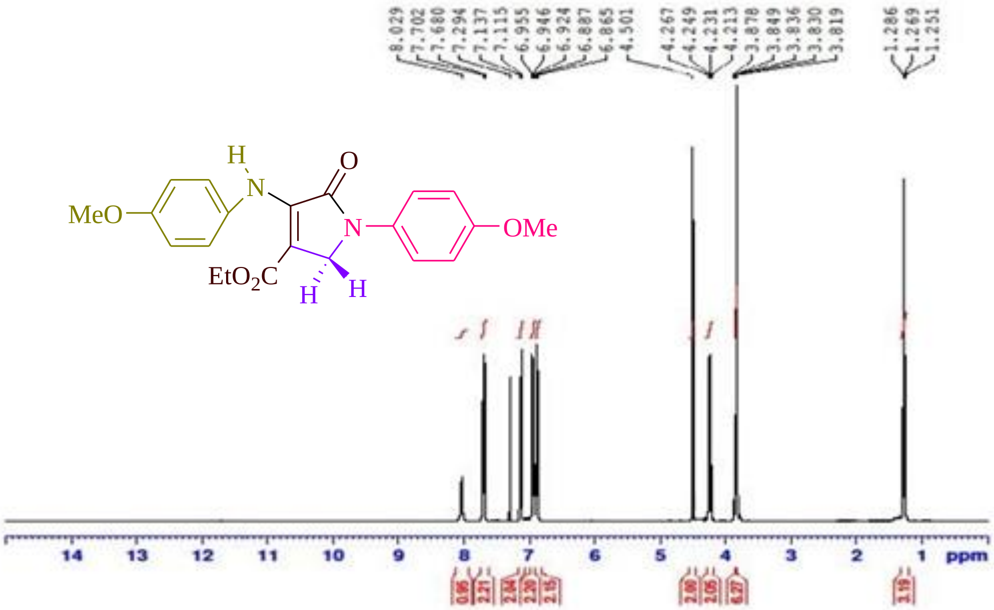

Fig. S22 ^{1}HNMR Spectrum of compound (400 MHz, $CDCl_3$) of **5r**

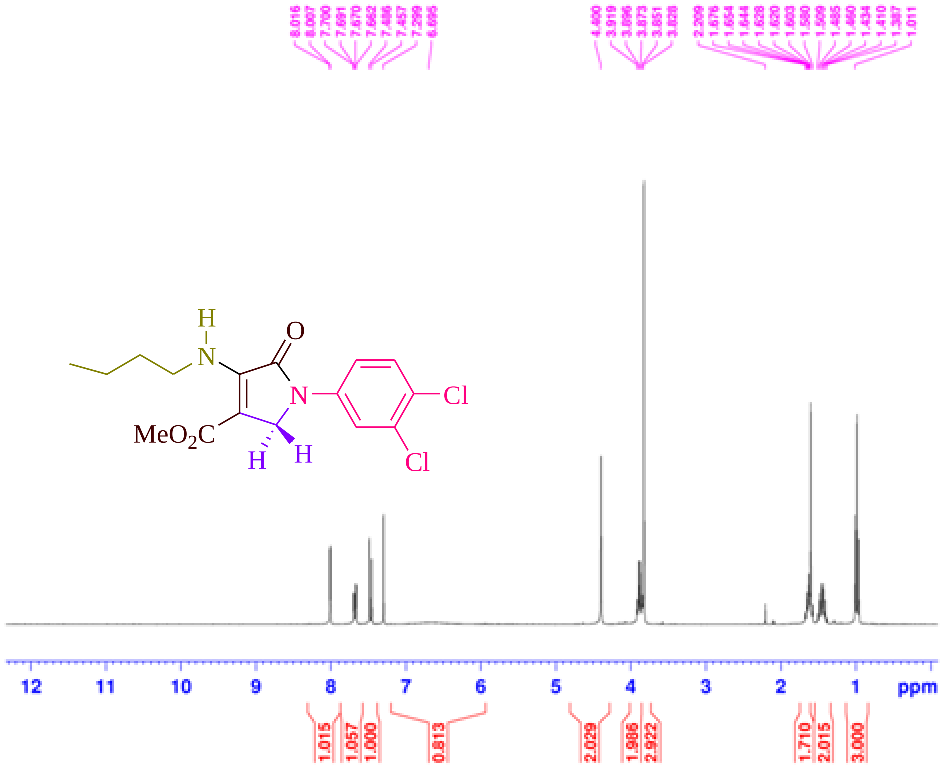

Fig. S23 ^{1}HNMR Spectrum of compound (300 MHz, $CDCl_3$) of **5s**
